# Real-world experience of CPX-351 as first-line treatment for patients with acute myeloid leukemia

**DOI:** 10.1038/s41408-021-00558-5

**Published:** 2021-10-04

**Authors:** Christina Rautenberg, Friedrich Stölzel, Christoph Röllig, Matthias Stelljes, Verena Gaidzik, Michael Lauseker, Oliver Kriege, Mareike Verbeek, Julia Marie Unglaub, Felicitas Thol, Stefan W. Krause, Mathias Hänel, Charlotte Neuerburg, Vladan Vucinic, Christian-Friedrich Jehn, Julia Severmann, Maxi Wass, Lars Fransecky, Jens Chemnitz, Udo Holtick, Kerstin Schäfer-Eckart, Josephine Schröder, Sabrina Kraus, William Krüger, Ulrich Kaiser, Sebastian Scholl, Kathrin Koch, Lea Henning, Guido Kobbe, Rainer Haas, Nael Alakel, Maximilian-Alexander Röhnert, Katja Sockel, Maher Hanoun, Uwe Platzbecker, Tobias A. W. Holderried, Anke Morgner, Michael Heuser, Tim Sauer, Katharina S. Götze, Eva Wagner-Drouet, Konstanze Döhner, Hartmut Döhner, Christoph Schliemann, Johannes Schetelig, Martin Bornhäuser, Ulrich Germing, Thomas Schroeder, Jan Moritz Middeke

**Affiliations:** 1 Department of Hematology, Oncology and Clinical Immunology, University Hospital Duesseldorf, Medical Faculty, Heinrich Heine—University, Duesseldorf, Germany; 2 Medizinische Klinik und Poliklinik I, University Hospital Carl Gustav Carus Dresden, Technical University Dresden, Dresden, Germany; 3 Department of Medicine A, University Hospital Münster, Münster, Germany; 4 Department of Internal Medicine III, University Hospital of Ulm, Ulm, Germany; 5 Institute for Medical Information Processing, Biometry and Epidemiology, Ludwig-Maximilians-University, Munich, Germany; 6 Department of Medicine III, University Medical Center, Johannes Gutenberg University Mainz, Mainz, Germany; 7 Department of Medicine III, Technical University of Munich, Munich, Germany; 8 Department of Internal Medicine V, University Hospital Heidelberg, Heidelberg, Germany; 9 Department of Hematology, Hemostasis, Oncology and Stem Cell Transplantation, Hannover Medical School, Hannover, Germany; 10 Department V for Internal Medicine, University Hospital Erlangen, Erlangen, Germany; 11 Department of Internal Medicine III, Klinikum Chemnitz, Chemnitz, Germany; 12 Department of Oncology, Hematology and Rheumatology, University Hospital Bonn, Bonn, Germany; 13 Leipzig: Department of Hematology and Cell Therapy, Medical Oncology, Hemostaseology, Leipzig, Germany; 14 Department of Hematology, Oncology and Stem Cell Transplantation, Asklepios Clinic St. Georg, Hamburg, Germany; 15 Department of Hematology and Stem Cell Transplantation, West German Cancer Center Essen, University Hospital Essen, Essen, Germany; 16 Clinic and Policlinic for Internal Medicine IV, University Hospital Halle (Saale), Halle (Saale), Germany; 17 Departmenf for Internal Medicine II, University Schleswig-Holstein, Kiel, Germany; 18 Gemeinschaftsklinikum Mittelrhein GGmbH, Koblenz, Germany; 19 Department I of Internal Medicine, University Hospital of Cologne, Cologne, Germany; 20 Department of Internal Medicine V, Oncology and Hematology, Klinikum Nürnberg, Nürnberg, Germany; 21 Clinic for Heaematology and Stem Cell Transplantation HELIOS Clinic Berlin-Buch GmbH, Berlin, Germany; 22 Division of Hematology and Oncology, Department of Internal Medicine II, University of Würzburg, Medical Center, Würzburg, Germany; 23 Clinic and Policlinic for Internal Medicine C, Hematology and Oncology, University of Greifswald, Greifswald, Germany; 24 Department of Hematology and Oncology, St. Bernward Krankenhaus, Hildesheim, Germany; 25 Department of Internal Medicine II, Hematology and Oncology, University Hospital Jena, Jena, Germany

**Keywords:** Haematological cancer, Leukaemia

## Abstract

To investigate the efficacy and toxicities of CPX-351 outside a clinical trial, we analyzed 188 patients (median age 65 years, range 26–80) treated for therapy-related acute myeloid leukemia (t-AML, 29%) or AML with myelodysplasia-related changes (AML-MRC, 70%). Eighty-six percent received one, 14% two induction cycles, and 10% received consolidation (representing 22% of patients with CR/CRi) with CPX-351. Following induction, CR/CRi rate was 47% including 64% of patients with available information achieving measurable residual disease (MRD) negativity (<10^−3^) as measured by flow cytometry. After a median follow-up of 9.3 months, median overall survival (OS) was 21 months and 1-year OS rate 64%. In multivariate analysis, complex karyotype predicted lower response (*p* = 0.0001), while pretreatment with hypomethylating agents (*p* = 0.02) and adverse European LeukemiaNet 2017 genetic risk (*p* < 0.0001) were associated with lower OS. Allogeneic hematopoietic cell transplantation (allo-HCT) was performed in 116 patients (62%) resulting in promising outcome (median survival not reached, 1-year OS 73%), especially in MRD-negative patients (*p* = 0.048). With 69% of patients developing grade III/IV non-hematologic toxicity following induction and a day 30-mortality of 8% the safety profile was consistent with previous findings. These real-world data confirm CPX-351 as efficient treatment for these high-risk AML patients facilitating allo-HCT in many patients with promising outcome after transplantation.

## Introduction

Acute myeloid leukemia (AML) can arise de novo, as therapy-related complication following chemotherapy and/or ionizing radiation (t-AML) or from antecedent hematologic disorders [1]. The latter are also summarized as secondary AML (sAML) and account for approximately one quarter of all AML cases. AML with myelodysplasia-related changes (AML-MRC) is defined according to the WHO 2016 classification by the history of a myelodysplastic syndrome (MDS), signs of dysplasia, and/or MDS-related cytogenetic abnormalities. AML-MRC and sAML occur more frequently with advanced age and are associated with biologic properties such as adverse genetics and multidrug resistance phenotype, which contribute to poor outcome after conventional therapy [2–5]. While attempts to improve outcome after induction therapy by addition of other agents or intensification of post-remission therapy have generally failed, higher remission rates and longer overall survival (OS) compared to conventional cytarabine plus daunorubicin chemotherapy (7 + 3 regimen) were recently observed following CPX-351 (Jazz Pharmaceuticals, Palo Alto, CA), a liposomal encapsulation of cytarabine and daunorubicin at a fixed 5:1 synergistic molar ratio [6, 7]. The results from this phase-III trial, which investigated CPX-351 in 309 patients with AML-MRC or tAML aged 60–75 years, led to the approval of the drug combination by the health authorities in the USA 2017 and in Europe 2018 for adult patients with newly diagnosed AML-MRC or t-AML. Beside the aspect of age also several other issues, e.g. measurable residual disease (MRD), molecular subgroups, specific side effects as well as specific outcome parameters in the context of allogeneic hematopoietic cell transplantion (allo-HCT) were not addressed in the phase-III trial. Furthermore, in other settings data from real-world experiences with new therapies differed from clinical trial results [8, 9], suggesting that patients in clinical trials represent a selected cohort potentially limiting interpretation and translation of results to real-life patient care. Two recent retrospective analyses pointed towards some of these open questions [10, 11]. Therefore, aiming to address these open aspects and to provide more clinical data and experience for CPX-351, we performed a real-world analysis of consecutive newly diagnosed patients with AML, who were treated in-label with CPX-351 as first-line therapy.

## Subjects and methods

### Study design

For this retrospective analysis, we collected data from consecutive patients with newly diagnosed AML-MRC or t-AML, who were treated with CPX-351 according to the EMA label between 2018 and 2020 in 25 German centers participating in the Study Alliance Leukemia (SAL), German Cooperative Transplant Study Group, and the AML Study Group (AMLSG). Median number of patients included per center was 6 (range, 1–22 patients). Information about patient characteristics, treatment details including allo-HCT, and outcome were gathered using a specific, standardized data form sent to the participating centers. All patients in the participating centers treated with CPX-351 as first-line therapy for AML during this time period were reported. To ensure high data quality physicians’ review of data and personal requests at respective centers was performed for all patients. The study was approved by the ethics committee of the Heinrich-Heine-University, Duesseldorf (approval number: 2020-877) and all patients gave written informed consent for scientific use of their data. Data lock for the analysis was 1 November 2020.

### Definitions and response criteria

AML subtypes were categorized according to the criteria of the WHO 2016 classification [1]. Response, relapse, and genetic risk categories were defined according to the 2017 European LeukemiaNet (ELN) recommendations [12]. For the definition of complete remission without minimal residual disease (CR_MRD−_) we used information of MRD results obtained by routine flow cytometry (FC)-based MRD monitoring performed at the participating site.

Conditioning intensity, the hematopoietic cell transplantation-specific comorbidity index (HCT-CI), and graft-versus-host disease (GvHD) were defined as previously described [13–16]. Non-hematologic toxicity was graded by the treating physician using the National Cancer Institute Common Toxicity Criteria (NCI CTC).

### Statistical analyses

For categorical variables frequencies were displayed and differences were evaluated using cross-tabulation and Fisher’s exact *t*-test, whereas for continuous variables medians (range) were summarized and the Mann–Whitney test was used to detect differences. OS for the entire cohort was calculated as the time from the first day of treatment with CPX-351 to death from any cause or last follow-up in survivors. For the subgroup of patients undergoing allo-HCT OS was estimated as time between allo-HCT and death or date of last follow-up in surviving patients, while relapse-free survival (RFS) was calculated as time from allo-HCT until relapse or death without relapse censoring those patients, who had not relapsed until and were alive at date of last follow-up. Time-to-event curves were calculated by employing the Kaplan–Meier method and log-rank tests were applied for univariate comparisons.

Furthermore, for the transplant cohort cumulative incidence of relapse (CIR) and non-relapse mortality (NRM) were considered as competing risks and calculated using cumulative incidence (CI) estimates employing Gray test for univariate comparisons. Those parameters, which influenced OS of the entire cohort in univariate analysis with *p* < 0.10, were included into a multivariate analysis using a proportional hazard regression analysis (multiple Cox regression model). For factors associated with achievement of CR/CRi a multinominal logistic regression analysis was applied.

In all analyses, a *p* value <0.05 was considered to be significant. Statistical analyses were performed using GraphPad Prism® 7 (GraphPad Software Inc., La Jolla, USA) and IBM SPSS Statistics (SPSS Inc. Chicago, IL) as well as R 3.5.1.

## Results

### Patients characteristics

We analyzed data from 188 consecutive patients, who received CPX-351 induction chemotherapy as first-line therapy for AML between June 2018 and June 2020. Median age was 65 years (range 26–80 years) and 46 patients (24%) were <60 years. Most patients (82%) had a good performance status as indicated by a Karnofsky index of ≥80%, while the majority (82%) exhibited comorbidities as indicated by intermediate or high HCT-CI. A diagnosis of t-AML was present in 29% of patients, while 70% were diagnosed with AML-MRC. A total of 19 patients (10%) had been treated with hypomethylating agents (HMA) for anteceding MDS before initiation of CPX-351. Karyotype abnormalities were present in 65% of patients including 44 patients (25%) with a complex karyotype (CK). Considering the prognostically relevant mutations included in the 2017 ELN risk classification, *ASXL1* (*n* = 31, 16%) was the most frequently mutated gene, followed by *RUNX1* (*n* = 24, 13%), *NPM1* (*n* = 18, 10%), *TP53* (*n* = 14, 7%), and *FLT3*-ITD (*n* = 13, 7%). Accordingly, 7, 33, and 60% of patients belonged to the favorable, intermediate, and adverse ELN 2017 genetic risk category, respectively. Detailed patients characteristics are summarized in Table 1.

**Table 1:** Patient characteristics (*n* = 188).

Characteristics	*n*	%
Age, years (median, range)	65 (26–80)	
Gender		
Female	70	37
Male	118	63
Karnofsky (*n* = 165)		
≥80	135	82
<80	30	18
HCT-CI (*n* = 155)		
Low	29	18
Intermediate	55	35
High	74	47
AML subtype (*n* = 186)		
AML-MRC	131	70
t-AML	53	29
Other^a^	2	1
2017 ELN genetic risk (*n* = 179)		
Favorable	12	7
Intermediate	59	33
Adverse	108	60
Karyotype (*n* = 179)		
Normal	63	35
Abnormal	116	65
Complex	44	25
Non-complex	72	40
Molecular genetics		
NPM1/n.d.	18/11	10/6
FLT3-ITD/n.d.	13/13	7/7
ASXL1/n.d.	31/24	16/13
TP53/n.d.	14/26	7/14
RUNX1/n.d.	24/20	13/11
BM blast count at diagnosis (median, range)	38 (7–99)	
PB blast count at diagnosis (median, range)	10 (0–92)	
WBC at diagnosis, ×10^3^/µl (median, range)	3.8 (0.6–330)	
No treatment prior CPX	169	90
Pretreatment with HMA	19	10

*AML* acute myeloid leukemia, *AML-MRC* AML with myelodysplasia-related changes, *BM* bone marrow, *ELN* European Leukemia Net, *HCT-CI* hematopoietic cell transplantation-specific comorbidity index, *HMA* hypomethylating agents, *n.d.* not done, *n* number, *PB* peripheral blood, *t-AML* therapy-related AML, *WBC* white blood cells.

Numbers in parentheses display patients with available information.

^a^Secondary AML evolving from systemic mastocytosis (*n* = 1) and blastic plasmacytoid dendritic cell neoplasm (BPDCN) with antecedent history of chronic myelomonocytic leukemia (CMML) (*n* = 1).

### Treatment

**Fig. 1:**
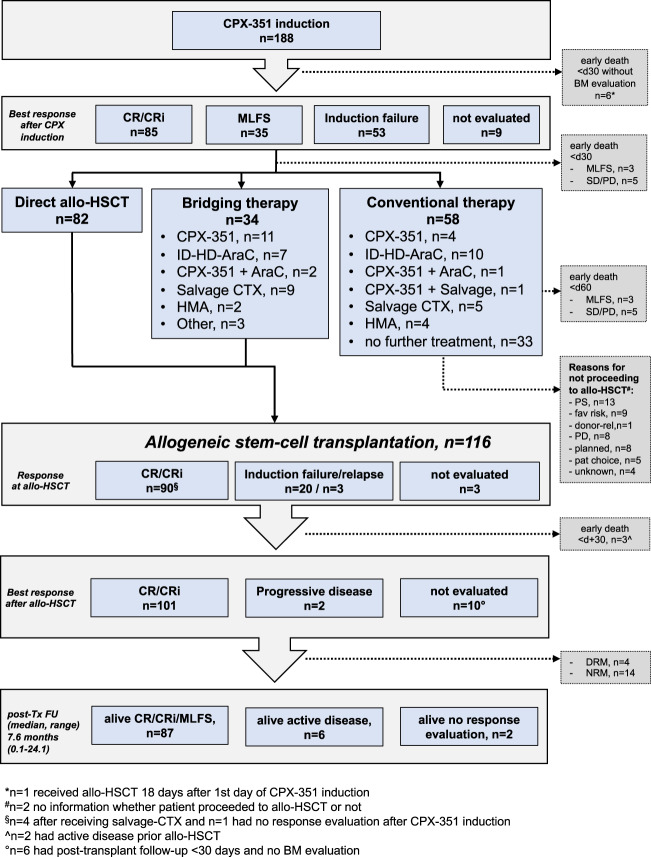
Flow chart depicting treatment, response, and outcome of the study population. Allo-HSCT allogeneic hematopoietic stem cell transplantation, BM bone marrow, CR complete remission, CRi complete remission with incomplete hematologic recovery, CTX chemotherapy, DRM disease-related mortality, d day, fav favorable, FU follow-up, HD-AraC high-dose cytarabine, ID-AraC intermediate dose cytarabin, MLFS morphologic leukemia-free state, NRM non-relapse mortality, pat patient, PD progressive disease, PS performance status, rel related, SD stable disease, Tx transplant.

One-hundred and sixty-two patients (86%) received one cycle of CPX-351 induction, while 26 patients (14%) received two induction cycles (Table 2). Of these, 116 (62%) underwent allo-HCT, which was performed without further therapy in 82 patients. In the remaining 34 patients at least one cycle of intermittent therapy consisting of either CPX-351 and/or AraC consolidation, HMA or salvage therapy was performed (Fig. 1). In 58 patients (31%), who were alive beyond day +30 after induction, no allo-HCT was performed. Reasons for not proceeding to allo-HCT were mainly related to performance status, favorable disease risk, progressive disease, or patients’ decision (Fig. 1). Of these non-transplanted patients, 25 patients received at least one other line of conventional therapy consisting of CPX-351 and/or AraC consolidation, HMA or salvage therapy, while in the remaining 33 patients no further therapy was performed (Fig. 1). Overall, at least one cycle of CPX-351 consolidation therapy was administered in 19 patients (10%) of patients, which corresponds to 22% of patients with CR/CRi.

**Table 2:** Treatment characteristics, response, and toxicity.

Characteristics	*n*	%
Follow-up, months (median, range)	9.3 (0.2–26.1)	
No. of induction cycles with CPX (median, range)	1 (1–2)	
ANC recovery^a^ in patients with CR/CRi after CPX-351 induction (*n* = 83)		
Yes	79	95
No	4	5
Time, days (median, range)	33 (6–99)	
Platelet recovery^b^ in patients with CR/CRi after CPX-351 induction (*n* = 83)		
Yes	76	92
No	7	8
Time, days (median, range)	30 (7–77)	
Response after induction (*n* = 179)		
CR/CRi	85	47
MLFS	35	20
Refractory disease	53	30
Early death (<30 days) without response	6	3
Evaluation		
MRD available in case of CR/CRi	36	42^c^
MRD negative	23	64^d^
Grade III/IV non-hematologic toxicities (*n* = 188)		
Infection	41	22
GI (mucositis, nausea, vomiting)	7	4
Bleeding	7	4
Renal failure	5	3
Febrile neutropenia	28	15
Pneumonia	42	22
Mortality on d30 after CPX-351 induction (*n* = 176)^e^		
Alive	162	92
Dead	14	8

*ANC* absolute neutrophile count, *CR* complete remission, *CRi* complete remission with incomplete hematologic recovery, *d* day, *GI* gastrointestinal, *MLFS* morphologic leukemia-free state, *MRD* minimal residual disease, *n* number, *SD* stable disease, *PD* progressive disease, *n.d.* not done.

Numbers in parentheses display patients with available information.

^a^Defined as ANC > 500/µl.

^b^Defined as platelet count >50,000/µl.

^c^Of the 85 patients with CR/CRi.

^d^Of the 36 patients with available MRD results.

^e^Patients proceeding to allo-HSCT before d30 following CPX-351 induction have been excluded.

### Response after induction

Following CPX-351- induction 47% of evaluable patients achieved CR/CRi (*n* = 85) and 20% (*n* = 35) morphologic leukemia-free state (MLFS), while 30% of evaluable patients (*n* = 53) did not respond. Among patients with CR/CRi (*n* = 85) after CPX-351 induction data on MRD measured by flow cytometry were available for 36 patients (42%) demonstrating MRD negativity in 64% of the patients (*n* = 23) (Table 2). CR/CRi rate in patients with complex karyotype (*n* = 44) and in those with TP53 mutation (*n* = 14) were 33% and 54%, respectively.

Given the limited data in patients treated with CPX-351 so far [6, 10, 11], we aimed to identify predictors for achievement of CR/CRi. In univariate analyses, we observed higher CR/CRi rates in female patients, in those without complex karyotype and in those patients without HMA pretreament (Supplementary Table 1). In multivariate analysis, only a non-complex karyotype (*p* = 0.0001) predicted for a higher CR/CRi rate (Table 3).

**Table 3:** Outcome after induction with CPX-351, multivariate analysis.

Variable	Overall survival			Response rate		
	*P*		HR	*P*		HR
**Prior treatment with HMA**						
Yes	0.02		2.4 [1.1–5.3]		n.s.	
No						
**ELN risk stratification**						
Adverse	<0.0001		4.2 [1.9–8.9]		–	
Favorable/intermediate						
**Karyotype**						
Complex		–		0.0001		4.3 [1.9–9.2]
Not complex						
***NPM1***						
wt		n.s.	–			
mut						
***TP53***						
mut		n.s.	–			
wt						
**Age at diagnosis (median)**						
≥65		n.s.	–			
<65						
**Gender**						
Female		–	n.s.			
Male						

*ELN* European Leukemia Net, *HMA* hypomethylating agents, *HR* hazard ratio, *mut* mutated, *n.s.* not significant, *P* *p* value, *wt* wild type.

### Safety

In patients with CR/CRi recovery to an absolute neutrophil count (ANC) ≥ 500/µl and platelet count ≥50.000/µl was observed in 95% and 92% of patients, respectively (Table 2). Median time to ANC and platelet recovery was 33 days (range: 6–99 days) and 30 days (range: 7–77 days), respectively.

Regarding non-hematologic toxicity, adverse events (AE) ≥ grade III were reported in 130 patients (69%). As indicated in Table 2, these were mainly related to infectious complications, while gastrointestinal side effects and bleeding occurred rather infrequently. Of note, patients with leukocyte counts >20 G/L at diagnosis had a significantly higher frequency of grade III/IV AE as compared to those with leukocytes <20 G/L (74% vs. 50%, *p* = 0.01). The 30-day early death rate was 8% in the entire cohort and was significantly higher in patients ≥ 65 years (11% vs. 3%, *p* = 0.047).

### Overall survival of the entire cohort

**Fig. 2:**
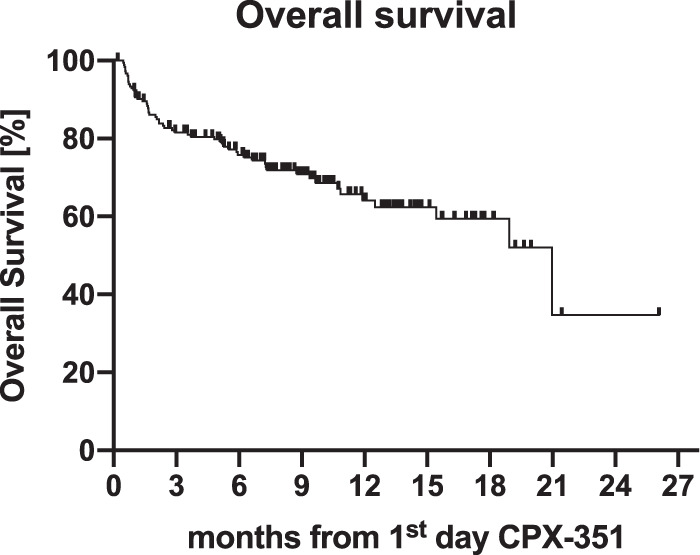
Overall (OS) of the entire cohort. After a median follow-up of 9.3 months (range: 0.2–26.1 months) median OS of the entire cohort (*n* = 188) was 21 months and estimated 2-year OS was 35%. OS for the entire cohort was calculated as the time from the first day of treatment with CPX-351 to death from any cause or last follow-up in survivors.

With a median follow-up (FU) of 9.3 months (range: 0.2–26.1 months) median OS of the entire cohort was 21 months and estimated 1-year OS was 64% (95% CI 55–72%, Fig. 2). In univariate analysis, pretreatment with HMA, the presence of an abnormal or complex karyotype, adverse ELN genetic risk, age ≥65 years, MRD positivity after induction, and not undergoing allo-HCT were associated with inferior OS (Supplementary Table 2). In multivariate analysis, pretreatment with HMA (*p* = 0.02) and adverse ELN genetic risk (*p* < 0.0001) retained their negative impact on OS (Table 3).

### Allo-HCT

One-hundred and sixteen (62%) patients underwent allo-HCT after CPX-351 induction with a median time from start of induction to transplantion of 70 days (range: 11–215 days). At the time of transplant 20% (*n* = 23) of patients had active disease (>5% BM blasts, induction failure *n* = 20, relapse *n* = 3), while 80% (*n* = 90) were in first remission (Fig. 1). The latter included 26 of the 35 patients initially categorized as MLFS at first remission control, which were classified as CRi (*n* = 16) or CR (*n* = 10) at the time of transplant. Among patients with CR/CRi at transplant flow-cytometry-measured MRD status was available in 36 patients with 23 patients (64%) exhibiting MRD negativity at the time of transplant. Detailed information on transplant characteristics are summarized in Table 4 and Supplementary Tables 3 and 4. The majority of patients (*n* = 95, 82%) received a graft from an unrelated donor following a reduced-intensity conditioning (RIC), which was used in all patients except for one.

**Table 4:** Transplant characteristics, toxicity and outcome.

Characteristics	*n*	%
Time between 1st day of CPX-351 and allo-HCT, days (median, range)	70 (11–215)	
Median age at transplant	64 (24–79)	
HLA-matching (*n* = 116)		
MUD	74	64
MRD	13	11
MMUD	21	18
Haploidentical	8	7
Donor gender (*n* = 112)		
Male	78	70
Female	34	30
CMV-status (donor/recipient) (*n* = 111)		
pos/pos	44	40
pos/neg	7	6
neg/neg	35	32
neg/pos	25	22
Conditioning intensity (*n* = 116)		
Reduced intensity	115	99
Standard dose	1	1
Stem cell source (*n* = 113)		
PBSC	110	97
BM	3	3
In vivo T cell depletion (*n* = 110)		
Yes	69	63
No	41	37
Engraftment (*n* = 114)		
No	2	2
Yes	112	98
ANC recovery after allo-HCT (*n* = 101)		
Yes	100	99
No	1	1
Time, days (median, range)	16 (7–70)	
Platelet recovery after allo-HCT (*n* = 101)		
Yes	96	96
No	5	5
Time, days (median, range)	15 (9–45)	
Acute GvHD (*n* = 113)		
Yes	56	50
No	57	50
≥Grade III	15	13
Chronic GvHD (*n* = 110)		
Yes	23	21
No	87	79
Severe	4	4
Grade III/IV non-hematologic toxicities (*n* = 116)		
Infection	36	31
GI (mucositis, nausea, vomiting)	11	9
Bleeding	2	2
Renal failure	8	7
Febrile neutropenia	2	2
SOS	1	1
Relapse after allo-HCT		
Yes	11	11
Time between allo-HCT and relapse, days (median, range)	82 (33–549)	
No	90	89
Posttransplant day + 100 mortality (*n* = 116)		
Dead	8	7
Alive	95	82
Alive, evaluated <day +100	13	11
Posttransplant follow-up, months (median, range)	7.6 (0.1–24.1)	

*ANC* absolute neutrophile count, *Allo-HCT* allogeneic hematopoietic cell transplantation, *BM* bone marrow, *CMV* cytomegalovirus, *GI* gastrointestinal, *GvHD* graft-versus-host disease, *HLA* human leukocyte antigen, *MMUD* mismatched unrelated donor, *MRD* matched related donor, *MUD* matched unrelated donor, *neg* negative, *PBSC* peripheral blood stem cells, *pos* positive, *SOS* sinusoidal obstruction syndrome.

Numbers in parentheses display patients with available information.

A primary graft failure was observed in two patients (2%). Median time to ANC and platelet recovery was 16 days (range: 7–70 days) and 15 days (range: 9–45 days), respectively. A sinusoidal obstruction syndrome (SOS) was reported in one patient (1%). Acute GvHD (aGvHD) occurred in 50% of patients and aGVHD ≥grade III was observed in 13% of patients, while chronic GvHD (cGvHD) was seen in 21% of patients including 4% with severe cGvHD. In evaluable patients, day 100 mortality was 7%.

**Fig. 3:**
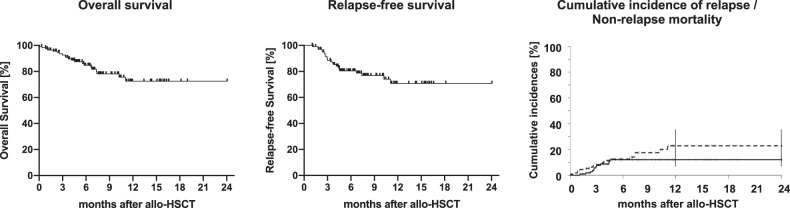
Overall (OS), relapse-free survival (RFS), cumulative incidences of relapse (CIR), and non-relapse mortality (NRM) of the transplant cohort. With a median FU of 7.6 months (range: 0.1–24.1 months) estimated 2-year OS, RFS, CIR, and NRM probabilities of the entire cohort were 73%, 71%, 23%, and 12%, respectively. OS was estimated as time between allo-HSCT and death or date of last follow-up in surviving patients, while RFS was calculated as time from allo-HSCT until relapse or death without relapse censoring those patients, who had not relapsed until and were alive at date of last follow-up. CIR and NRM were considered as competing risks and calculated using cumulative incidence (CI) estimates employing Gray test for univariate comparisons.

**Fig. 4:**
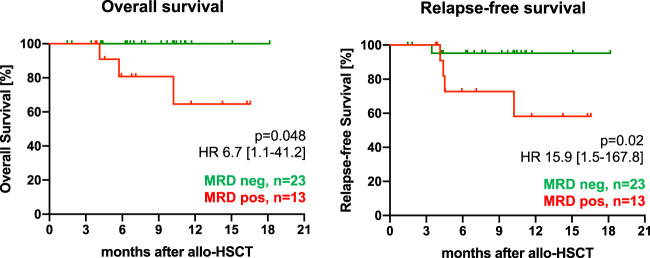
Posttransplant outcome of patients depending on pre-transplant MRD status assessed by flow cytometry (FC). Among patients with CR/CRi (*n* = 85) after CPX-351-based induction data on MRD estimated by FC were available for 36 patients (42%) representing MRD negativity in 64% of the patients (*n* = 23). The figure represents outcome in terms of overall and relapse-free survival for patients with pre-transplant CR_MRD−_ (*n* = 23, green line) and CR_MRD+_ (*n* = 13, orange line).

With a median FU of 7.6 months (range: 0.1–24.1 months) 1-year OS, RFS, CIR, and NRM probabilities 73%, 71%, 23%, and 12%, respectively (Fig. 3). In a next step, similar to the entire cohort, we aimed to identify predictors for outcome after allo-HCT. In univariate analyses, only MRD positivity prior to transplant was associated with shorter OS (Fig. 4), while diagnosis of t-AML and active disease at time of transplant adversely impacted RFS (Supplementary Table 5). A complex karyotype, previous treatment with HMA, and a mismatched donor were associated with a higher relapse incidence in univariate analyses (Supplementary Table 5). Due to the low number of events no parameter associated with NRM could be ascertained. Together with the limited availability of information regarding some parameters in all patients this also impeded multivariate analyses.

## Discussion

Here, we report data from 188 patients treated in-label with CPX-351 as first-line treatment for the diagnosis of AML. This patient number, which represents to the best of our knowledge the largest cohort outside a clinical trial reported so far, enabled us to provide sufficient data on response and outcome, course of treatment including allo-HCT and to identify predictors for response and survival.

First, our results show that in a real-life setting CPX-351 is used in patients exhibiting high-risk characteristics, which are in many aspects comparable to those in the phase-III trial, but also to those reported in two other real-world data sets of 103 and 71 patients [6, 10, 11]. This applies to the median age, performance status, comorbidities, frequency of AML subtypes, BM blast count, cytogenetics, molecular abnormalities, and NCCN/ELN risk categorization. Indeed, similar to the other cohorts [6, 10, 11] secondary-type mutations [17] in genes like *ASXL1*, *RUNX1,* and *TP53* mutations represented the most common alterations, but most interestingly neither influenced response or survival following CPX-351 treatment. This is in contrast to the pivotal and also the French study, in which at least *TP53* mutations predicted for inferior response. Still, it has to be taken into account that molecular data could not be comprehensively analyzed in all patients. The higher frequency of patients with HMA pretreatment in the prospective trial compared to the three real-world series might be related to the approval of HMA also for low-risk MDS in the US.

Despite many similarities in patients characteristics the treatment course substantially differed between our cohort, the population in the prospective trial, and the two real-world series [6, 10, 11]. Potentially due to the trial design a higher proportion of patients in the prospective study (31% compared to 5–14%) received a second induction with CPX-351. In contrast, in our cohort a lower frequency of patients received at least one CPX-351 consolidation (10%) compared to the three other populations (ranging from 32 to 46%). This is probably a consequence of a much higher rate of patients undergoing allo-HCT in our cohort (62% vs. 27–35%), with the majority proceeding directly to transplant after induction. The latter might be related to a higher frequency of rapidly available donors and/or a general treatment philosophy to proceed to transplant in as many patients as possible. Additionally, since a similar frequency of 20 patients in our cohort received conventional intermediate/high-dose cytarabine for consolidation, the low rate of CPX-351 consolidation might also be related in parts to a greater familiarness of physicians with conventional cytarabine consolidation.

Following induction, we observed an identical CR/CRi rate of 47% like in the prospective trial, while CR/CRi rates were 70 and 59% in the Italian and French patients [6, 10, 11]. Response assessment was not performed at a pre-defined time point in the retrospective analyses. Of note, from 35 patients initially classified as MLFS, 26 turned into CR/CRi at a later point. Thus, from these data a CR/CRi rate of 47% can realistically be expected after CPX-351 induction. If even a higher CR/CRi might be reached, will be prospectively addressed in the ongoing AMLSG 30-18 trial (NCT03897127).

The survival benefit of CPX-351 in the phase-III trial in all patients and those achieving CR/CRi but not undergoing allo-HCT as well as the likelihood of proceeding to allo-HCT suggest the potential for achievement of deeper responses with CPX-351 [6, 18]. While the impact of MRD was not prospectively addressed in this study, we observed MRD negativity detected by MFC in 64% of CR/CRi patients after induction. This appears not only similar to a rate of MRD negativity at the 10^−3^ sensitivity threshold (assessed with MFC or molecular methods) in 57% of patients of the French series [10], but also seems to be higher than the rate of MRD negativity observed after conventional 3 + 7 induction in such a high-risk population [19]. Accounting for the retrospective character and the limited patient number with available MRD information, the impact of MRD requires prospective and standardized investigation as envisaged, for example, in the AMLSG 30-18 trial.

The median survival of 21 and 16.1 months in our and the French cohort and the 1-year OS rate of 73% support the impression from the prospective trial that CPX-351 confers a survival benefit in such a high-risk population [6, 18]. Still, we have to acknowledge the short median follow-up of 9.3 months of our patients, which is comparable with the French (8.6 months) and Italian (11 months) cohorts, but definitively represents a limitation of our analysis.

Besides its efficacy in terms of remission induction, a favorable toxicity profile and a high rate of allo-HCT with favorable outcome after transplant contributed to the survival benefit in the phase-III trial. The low frequency of grade III/IV adverse events, especially gastrointestinal, expectable times to neutrophil and platelet recovery without excess of severe infectious complications and a day 30 mortality of 8% observed in our analysis confirm the favorable toxicity profile of CPX-351. This probably also contributed to the high rate of patients proceeding to allo-HCT with a RIC-based transplant. Indeed, we observed engraftment in all but two patients, hematopoietic recovery in the vast majority within an appropriate time, only one SOS and a low day 100 mortality of 7%. Together with a low relapse rate, especially in those patients transplanted in MRD-negative CR, this translated into a promising survival after transplant as already previously reported [6, 10, 11]. Finally, the opportunity of a growing number of treatment alternatives for patients with high-risk AML such as venetoclax [20] or glasdegib [21] renders the choice of optimal treatment more complex. Aiming to support physicians in this process, we identified previous HMA treatment and 2017 ELN adverse-risk categorization as negative predictors for survival and a complex karyotype for response in multivariate analysis. While in the prospective trial patients without HMA pretreatment also had also a survival benefit, this could not be supported by the results from the French and Italian consortia [6, 10, 11]. In the French cohort *TP53* and *PTPN11* mutations were associated with lower response rates, while splicosome mutations correlated with higher OS. In the Italian cohort no predictor for response could be ascertained, and performance of allo-HCT was the only factor associated with better survival. Therefore, the predictive and prognostic impact of patient- and disease-related factors including molecular characteristics in the context of CPX-351 treatment remains still elusive [22] and requires prospective investigation optimally within a randomized trial.

In summary, our data show that under real-world circumstances CPX-351 is an efficient and clinically relevant treatment option for patients with AML-MRC and t-AML. Given the promising outcome following transplant, a combined approach applying an effective induction with CPX-351 and inducing an MRD-negative CR followed by the subsequent allo-HCT without further delay [23] may constitute the treatment approach with the highest probability of cure for this high-risk population.

## Supplementary information

Supplemental Material

Reproducibility Checklist

Agreement Statement all authors
