# Supplementary material for: Real-world experience of CPX-351 as first-line treatment for patients with acute myeloid leukemia
Source: Blood Cancer J. 2021 Oct 4;11(10):164. doi: 10.1038/s41408-021-00558-5 (PMC8490353; doi:10.1038/s41408-021-00558-5)
Supplement: Supplementary file 1 — Supplemental Material [file 41408_2021_558_MOESM1_ESM.docx]

**Real-world experience of CPX-351 as first-line approach for patients with acute myeloid leukemia**

**Supplemental Material**

**Supplementary Tables:**

1. **Supplementary Table 1. Predictive factors for achievement of CR/CRi after induction with CPX-351, univariate analysis**
2. **Supplementary Table 2. Outcome after induction with CPX-351, univariate analysis**
3. **Supplementary Table 3. Conditioning regimen**
4. **Supplementary Table 4. Posttransplant Immunosuppression**
5. **Supplementary Table 5. Outcome after induction with CPX-351 and allo-HCT, univariate analysis**

**Supplementary Table 1. Predictive factors for achievement of CR/CRi after induction with CPX-351, univariate analysis**

| Variable | CR/CRi (%) | *P* |
| --- | --- | --- |
| Gender  female  male | 59  42 | **0.04** |
| Karnofsky Index  <80%  >80% | 60  46 | 0.27 |
| Type of AML  t-AML  AML-MRC | 53  48 | 0.61 |
| AML-MRC Subtype*  dysplasia  MDS-history  MDS-defining KT | 56  37  44 | 0.73  0.39  0.65 |
| Prior treatment with HMA  yes  no | 13  53 | **0.003** |
| ELN risk stratification  adverse  favorable/intermediate | 45  55 | 0.27 |
| Karyotype  complex  non-complex | 33  54 | **0.02** |
| Karyotype  abnormal  normal | 47  52 | 0.62 |
| *NPM1*  wt  mut | 48  59 | 0.44 |
| *FLT3*-ITD  wt  mut | 48  58 | 0.45 |
| *ASXL1*  mut  wt | 46  49 | 0.83 |
| *TP53*  mut  wt | 54  47 | 0.77 |
| *RUNX1*  mut  wt | 57  45 | 0.37 |
| Age (median)  >65  <65 | 45  55 | 0.36 |

AML, acute myeloid leukemia; AML-MRC, acute myeloid leukemia with MDS related changes; CR, complete remission; CRi, complete remission with incomplete hematologic recovery; ELN, European Leukemia Net; HMA, hypomethylating agents; KT, karyotype; MDS, myelodysplastic syndrome; mut, mutated; P, p-value; t-AML, therapy-related acute myeloid leukemia; wt, wild-type

*CR rates and p-values have to be interpreted as results of a comparison of the given subtype against the two other subtypes.

**Supplementary Table 2. Outcome after induction with CPX-351, univariate analysis**

| Variable | Overall survival  *P* HR | |
| --- | --- | --- |
| Gender  female  male | 0.79 | 1.1 [0.6-1.8] |
| Karnofsky Index  <80%  >80% | 0.16 | 1.6 [0.7-3.5] |
| Type of AML  t-AML  AML-MRC | 0.96 | 1.0 [0.6-1.8] |
| AML-MRC subtype*  dysplasia  MDS-history  MDS-defining KT | 0.06  0.99  0.21 | 0.3 [0.1-1.1]  1.0 [0.5-2.1]  1.7 [0.8-3.8] |
| Prior treatment with HMA  yes  no | **0.02** | 2.3 [0.9-6.1] |
| ELN risk stratification  adverse  favorable/intermediate | **<0.0001** | 3.5 [2.1-5.9] |
| Karyotype  complex  non-complex | **<0.01** | 2.2 [1.1-4.2] |
| Karyotype  normal  abnormal | **0.01** | 2.3 [1.3-4.0] |
| *NPM1*  wt  mut | 0.09 | 3.1 [1.3-7.4] |
| *FLT3*-ITD  mut  wt | 0.21 | 0.4 [0.2-1.1] |
| *ASXL1*  mut  wt | 0.64 | 1.2 [0.6-2.4] |
| *TP53*  mut  wt | 0.07 | 2.1 [0.7-5.9] |
| *RUNX1*  mut  wt | 0.93 | 1.1 [0.5-2.3] |
| Age at diagnosis (median)**  >65  <65 | **<0.01** | 2.2 [1.3-3.7] |
| Flow MRD  pos  neg | **0.01** | 9.3 [1.7-49.8] |
| Allo-HCT  no  yes | **<0.0001** | 5.1 [2.8-9.3] |

allo-HCT, allogeneic hematopoietic cell transplantation; AML, acute myeloid leukemia; AML-MRC, acute myeloid leukemia with MDS-related changes; ELN, European Leukemia Net; HMA, hypomethylating agents; HR, hazard ratio; KT, karyotype; MDS, myelodysplastic syndrome; MRD, minimal residual disease; mut, mutated; neg, negative; P, p-value; pos, positive; t-AML, therapy-related acute myeloid leukemia; wt, wild-type.

*HR and p-values have to be interpreted as results of a comparison of the given subtype against the two other subtypes.

**A significantly higher proportion of patients <65 years (75%) underwent allo-HSCT in comparison to patients > 65 years (50%, p<.001).

**Supplementary Table 3. Conditioning regimen**

| Conditioning regimen (n=111) | No. | % |
| --- | --- | --- |
| FLAMSA-based | 15 | 13 |
| Flu/Bu2 | 8 | 7 |
| Flu/Mel-based* | 15 | 13 |
| Flu/TBI 8Gy | 8 | 7 |
| Flu/Treo | 42 | 38 |
| Flu-BCNU-Mel | 9 | 9 |
| Other | 14 | 13 |

Bu, Busulfan; Flu, Fludarabin; Mel, Melphalan; No., number; TBI, total body irradiation; Treo, Treosulfan

*consisted of either Flu-Mel, Flu-Mel-Thiotepa, Flu-Mel-Treosulfan or Flu-Mel-TBI 8 Gy

Numbers in parentheses display patients with available information.

**Supplementary Table 4. Posttransplant Immunosuppression**

| Immunosuppression (n=107) | No. | % |
| --- | --- | --- |
| CSA/MMF | 46 | 43 |
| CSA/MTX | 34 | 32 |
| Tac/MMF | 15 | 14 |
| PtCy/Tac | 7 | 6 |
| PtCy/CSA | 3 | 3 |
| Other | 2 | 2 |
| In-vivo T-cell depletion (n=110)  yes  no | 69  41 | 63  37 |

CSA, Ciclosporin A; MMF, Mycophenolatmofetil; MTX, Methotrexate; No., number; PtCy, post-transplant Cyclophosphamide; Tac; Tacrolimus

Numbers in parentheses display patients with available information.

**Supplementary Table 5. Outcome after induction with CPX-351 and allo-HCT, univariate analysis**

| Variable | OS  *P* HR | | RFS  *P* HR | | CIR | |
| --- | --- | --- | --- | --- | --- | --- |
|  |  |  |  |  | *P* HR | |
| Gender  female  male | 0.71 | 0.8  [0.3-2.1] | 0.54 | 1.3  [0.6-3.0] | 0.26 | 0.5  [0.1-1.6] |
| Age at diagnosis (median)  >65  <65 | 0.08 | 2.3  [0.9-6.0] | 0.52 | 1.4  [0.6-3.4] | 0.68 | 0.8  [0.2-2.6] |
| HCT-CI  high  low/int | 0.63 | 1.3  [0.5-3.3] | 0.84 | 0.9  [0.4-2.1] | 0.94 | 1.0  [0.3-3.2] |
| Type of AML  t-AML  AML-MRC | 0.42 | 1.5  [0.5-4.3] | 0.03 | 2.4  [0.9-6.2] | 0.05 | 3.04  [1.0-9.4] |
| ELN risk stratification  adverse  fav/int | 0.05 | 2.8  [1.1-7.1] | 0.13 | 1.9  [0.9-4.5] | 0.44 | 1.6  [0.5-5.3] |
| Karyotype  complex  non-complex | 0.78 | 1.2  [0.4-3.8] | 0.26 | 1.7  [0.6-5.0] | 0.01 | 4.1  [1.3-12.6] |
| Karyotype  normal  abnormal | 0.12 | 2.4  [0.9-6.2] | 0.06 | 2.5  [1.1-5.8] | 0.06 | 6.9  [0.9-53.6] |
| Prior treatment with HMA  yes  no | 0.8 | 1.1  [0.3-4.2] | 0.22 | 1.8  [0.5-6.2] | 0.03 | 4.1  [1.1-15.2] |
| Remission status  remission  no remission | 0.18 | 2.0  [0.6-7.1] | 0.03 | 2.6  [0.8-8.2] | 0.11 | 2.7  [0.8-8.9] |
| Flow MRD  pos  neg | 0.02 | 15.9  [1.5-167.9] | 0.05 | 6.7  [1.1-41.2] | 0.29 | 3.6  [0.3-39.6] |
| HLA-matching  mismatched  matched | 0.81 | 0.8  [0.2-3.3] | 0.29 | 1.6  [0.6-4.8] | 0.02 | 3.7  [1.2-11.6] |
| Donor  unrelated  related | 0.83 | 1.1  [0.3-3.8] | 0.65 | 0.8  [0.3-2.3] | 0.87 | 1.1  [0.2-5.2] |

allo-HCT, allogeneic hematopoietic cell transplantation; AML, acute myeloid leukemia; AML-MRC, acute myeloid leukemia with MDS-related changes; CIR, cumulative incidence of relapse; ELN, European Leukemia Net; fav, favorable; HCT-CI, hematopoietic cell transplantation-specific comorbidity index; HLA, human leukocyte antigen; HMA, hypomethylating agents; HR, hazard ratio; int, intermediate; MRD, minimal residual disease; neg, negative; OS, overall survival; P, p-value; pos, positive; RFS, relapse-free survival; t-AML, therapy-related acute myeloid leukemia
