# Supplementary material for: Real-world experience of CPX-351 as first-line treatment for patients with acute myeloid leukemia
Source: Blood Cancer J. 2021 Oct 4;11(10):164. doi: 10.1038/s41408-021-00558-5 (PMC8490353; doi:10.1038/s41408-021-00558-5)
Supplement: Supplementary file 3 — Agreement Statement all authors [file 41408_2021_558_MOESM3_ESM.pdf]

Von: Hövelmann, Julia <Julia.Hoevelmann@uk-essen.de>
Gesendet: Donnerstag, 16. September 2021 01:22
An: Schroeder, Thomas <Thomas.Schroeder@uk-essen.de>
Betreff: AW: CPX-351 Paper Bitte um kurzfristige Rückmeldung

Please request agreement from all authors including additions and deletions, these can be collected in the following way:
Email your co-authors with the change, and ask them to reply to your email confirming that they agree to these changes. Once you have collected these replies, please combine all of the co-authors' email responses in one document and upload this file to your submission.

Agreed.

Herzliche Grüße,

Julia Hövelmann

Dr. med. Julia Hövelmann
Assistenzärztin
Klinik für Hämatologie und Stammzelltransplantation
Universitätsklinikum Essen

Please request agreement from all authors including additions and deletions, these can be collected in the following way:
Email your co-authors with the change, and ask them to reply to your email confirming that they agree to these changes. Once you have collected these replies, please combine all of the co-authors' email responses in one document and upload this file to your submission.

Agreed!

Best regards

Prof. Dr. med. Ulrich Kaiser
Med. Klinik II
St. Bernward Krankenhaus
Treibestraße 9
31134 Hildesheim
Telefon: 0 51 21 / 90 12 74
Telefax: 0 51 21 / 90 12 82
Email: med2@bernward-khs.de
Homepage: www.bernward-khs.de

Von: Henning, Lea <Lea.Henning@uk-essen.de>
Gesendet: Samstag, 11. September 2021 13:38

An: Alakel, Nael; Schroeder, Thomas; 'Kobbe@med.uni-duesseldorf.de'; Hanoun, Maher; 'Germing@med.uni-duesseldorf.de'; 'Haas@med.uni-duesseldorf.de'; 'Matthias.Stelljes@ukmuenster.de'; 'lauseker@ibe.med.uni-muenchen.de'; Schetelig, Johannes; Bornhäuser, Martin; 'Christoph.Schliemann@ukmuenster.de'; 'tim.sauer@med.uni-heidelberg.de'; 'heuser.michael@mh-hannover.de'; 'Döhner Hartmut'; 'Gaidzik Verena'; 'thol.felicitas@mh-hannover.de'; 'tobias.holderried@ukbonn.de'; 'eva.wagner@unimedizin-mainz.de'; 'oliver.kriege@unimedizin-mainz.de'; 'katharina.goetze@mri.tum.de'; 'mareike.verbeek@mri.tum.de'; 'a.morgner@skc.de'; 'Döhner Konstanze'; 'uwe.platzbecker@medizin.uni-leipzig.de'; 'Vladan'; Sockel, Katja; 'william.krueger@uni-greifswald.de'; 'stefan.krause@uk-erlangen.de'; 'udo.holtick@uk-koeln.de'; 'Kerstin.Schaefer-Eckart@klinikum-nuernberg.de'; 'Josephine.Schroeder@helios-gesundheit.de'; 'innere4@uk-halle.de'; 'Lars.Fransecky@uksh.de'; 'kmt-anfrage@ukw.de'; HIBK-Sekretariat Med2; 'charlotte.neuerburg@ukbonn.de'; 'Sebastian.Scholl@med.uni-jena.de'; 'c.jehn@asklepios.com'; 'nadine.scheer-wiaczka@gk.de'; 'jens.chemnitz@gk.de'; 'm.haenel@skc.de'; 'julia.unglaub@med.uni-heidelberg.de'
Cc: Rautenberg, Christina; Stölzel, Friedrich; Middeke, Jan Moritz
Betreff: [EXTERN] AW: CPX-351 Paper Bitte um kurzfristige Rückmeldung

Please request agreement from all authors including additions and deletions, these can be collected in the following way:
Email your co-authors with the change, and ask them to reply to your email confirming that they agree to these changes. Once you have collected these replies, please combine all of the co-authors' email responses in one document and upload this file to your submission.

Agreed.
Viele Grüße,
Lea Henning

Von: Alakel, Nael [Nael.Alakel@uniklinikum-dresden.de]
Gesendet: Freitag, 10. September 2021 07:51
An: Schroeder, Thomas; 'Kobbe@med.uni-duesseldorf.de'; Hanoun, Maher; 'Germing@med.uni-duesseldorf.de'; 'Haas@med.uni-duesseldorf.de'; 'Matthias.Stelljes@ukmuenster.de'; 'lauseker@ibe.med.uni-muenchen.de'; Schetelig, Johannes; Bornhäuser, Martin; 'Christoph.Schliemann@ukmuenster.de'; 'tim.sauer@med.uni-heidelberg.de'; 'heuser.michael@mh-hannover.de'; 'Döhner Hartmut'; 'Gaidzik Verena'; 'thol.felicitas@mh-hannover.de'; 'tobias.holderried@ukbonn.de'; 'eva.wagner@unimedizin-mainz.de'; 'oliver.kriege@unimedizin-mainz.de'; 'katharina.goetze@mri.tum.de'; 'mareike.verbeek@mri.tum.de'; 'a.morgner@skc.de'; 'Döhner Konstanze'; 'uwe.platzbecker@medizin.uni-leipzig.de'; 'Vladan'; Sockel, Katja; 'william.krueger@uni-greifswald.de'; 'stefan.krause@uk-erlangen.de'; 'udo.holtick@uk-koeln.de'; 'Kerstin.Schaefer-Eckart@klinikum-nuernberg.de'; 'Josephine.Schroeder@helios-gesundheit.de'; 'innere4@uk-halle.de'; 'Lars.Fransecky@uksh.de'; 'kmt-anfrage@ukw.de'; 'med2@bernward-khs.de'; 'charlotte.neuerburg@ukbonn.de'; 'Sebastian.Scholl@med.uni-jena.de'; 'c.jehn@asklepios.com'; 'nadine.scheer-wiaczka@gk.de'; 'jens.chemnitz@gk.de'; 'm.haenel@skc.de'; 'julia.unglaub@med.uni-heidelberg.de'; 'Germing@med.uni-duesseldorf.de'; 'Matthias.Stelljes@ukmuenster.de'; Henning, Lea
Cc: Rautenberg, Christina; Stölzel, Friedrich; Middeke, Jan Moritz
Betreff: AW: CPX-351 Paper Bitte um kurzfristige Rückmeldung

Please request agreement from all authors including additions and deletions, these can be collected in the following way:
Email your co-authors with the change, and ask them to reply to your email confirming that they agree to these changes. Once you have collected these replies, please combine all of the co-authors' email responses in one document and upload this file to your submission.

Agreed

Best regards
Nael Alakel

Dr.med. Nael Alakel
Funktionsoberarzt
Medizinischen Klinik und Poliklinik I.
Tel. +49 (0)351 458-2321
Fax +49 (0)351 458-5344

Universitätsklinikum Carl Gustav Carus
an der Technischen Universität Dresden
Anstalt des öffentlichen Rechts des Freistaates Sachsen
Fetscherstraße 74, 01307 Dresden
www.uniklinikum-dresden.de<http://www.uniklinikum-dresden.de>
Vorstand: Prof. Dr. med. D. Michael Albrecht (Sprecher), Frank Ohi
Vorsitzender des Aufsichtsrates: Univ.-Doz. Dr. G. Brunner
USt.-IDNr.: DE 140 135 217, St.-Nr.: 203 145 03113

Von: PD Dr. Michael Lauseker <lauseker@ibe.med.uni-muenchen.de>
Gesendet: Mittwoch, 15. September 2021 09:49
An: Schroeder, Thomas <Thomas.Schroeder@uk-essen.de>
Betreff: Re: CPX-351 Paper Bitte um kurzfristige Rückmeldung
Please request agreement from all authors including additions and deletions, these can be collected in the following way:
Email your co-authors with the change, and ask them to reply to your email confirming that they agree to these changes. Once you have collected these replies, please combine all of the co-authors' email responses in one document and upload this file to your submission.

Agreed

Lieber Herr Schroeder,
sorry für die späte Rückmeldung, ich war im Urlaub. Von meiner Seite gibt es natürlich keine Bedenken.
Gratulation zur Annahme!
Viele Grüße
Michael Lauseker

Von: Lars.Fransecky@uksh.de <Lars.Fransecky@uksh.de>
Gesendet: Montag, 13. September 2021 07:16
An: Schroeder, Thomas <Thomas.Schroeder@uk-essen.de>
Betreff: AW: CPX-351 Paper Bitte um kurzfristige Rückmeldung

Please request agreement from all authors including additions and deletions, these can be collected in the following way:
Email your co-authors with the change, and ask them to reply to your email confirming that they agree to these changes. Once you have collected these replies, please combine all of the co-authors' email responses in one document and upload this file to your submission.

Agreed!

Von: Schetelig, Johannes <Johannes.Schetelig@uniklinikum-dresden.de>
Gesendet: Samstag, 11. September 2021 11:32
An: Schroeder, Thomas <Thomas.Schroeder@uk-essen.de>
Betreff: Re: CPX-351 Paper Bitte um kurzfristige Rückmeldung

Please request agreement from all authors including additions and deletions, these can be collected in the following way:
Email your co-authors with the change, and ask them to reply to your email confirming that they agree to these changes. Once you have collected these replies, please combine all of the co-authors' email responses in one document and upload this file to your submission.

Agreed. Best, Johannes

Von: William Krüger <william.krueger@uni-greifswald.de>
Gesendet: Freitag, 10. September 2021 14:09
An: Schroeder, Thomas <Thomas.Schroeder@uk-essen.de>
Betreff: AW: CPX-351 Paper Bitte um kurzfristige Rückmeldung

Please request agreement from all authors including additions and deletions, these can be collected in the following way:
Email your co-authors with the change, and ask them to reply to your email confirming that they agree to these changes. Once you have collected these replies, please combine all of the co-authors' email responses in one document and upload this file to your submission.

Agreed!

Best regards, William Krüger

(Meine erstes ‚agreed' kam z. T. zurück)

-----Ursprüngliche Nachricht-----
Von: Charlotte.Neuerburg@ukbonn.de <Charlotte.Neuerburg@ukbonn.de>
Gesendet: Freitag, 10. September 2021 13:41
An: Schroeder, Thomas <Thomas.Schroeder@uk-essen.de>
Betreff: Antwort: CPX-351 Paper Bitte um kurzfristige Rückmeldung

Please request agreement from all authors including additions and deletions, these can be collected in the following way:
Email your co-authors with the change, and ask them to reply to your email confirming that they agree to these changes. Once you have collected these replies, please combine all of the co-authors' email responses in one document and upload this file to your submission.

Agreed

Dr. med. Charlotte Neuerburg
Assistenzärztin
Medizinische Klinik und Poliklinik III
Innere Medizin mit den Schwerpunkten
Onkologie, Hämatologie und Rheumatologie Sigmund-Freud-Str. 25
53127 Bonn

Von: jehn christian <cjehn2016@gmail.com>
Gesendet: Freitag, 10. September 2021 12:39
An: Schroeder, Thomas <Thomas.Schroeder@uk-essen.de>
Betreff: Re: [EXTERN] CPX-351 Paper Bitte um kurzfristige Rückmeldung
Priorität: Hoch

Please request agreement from all authors including additions and deletions, these can be collected in the following way:
Email your co-authors with the change, and ask them to reply to your email confirming that they agree to these changes. Once you have collected these replies, please combine all of the co-authors' email responses in one document and upload this file to your submission.

Agreed!

Viele Grüße

Christian Jehn

Von: Platzbecker, Uwe <Uwe.Platzbecker@medizin.uni-leipzig.de>
Gesendet: Freitag, 10. September 2021 11:08
An: Schroeder, Thomas <Thomas.Schroeder@uk-essen.de>
Betreff: WG: [Extern] - CPX-351 Paper Bitte um kurzfristige Rückmeldung

Please request agreement from all authors including additions and deletions, these can be collected in the following way:
Email your co-authors with the change, and ask them to reply to your email confirming that they agree to these changes. Once you have collected these replies, please combine all of the co-authors' email responses in one document and upload this file to your submission.

ok

HG
U. Platzbecker

Von: Wagner, Eva-Maria <Eva.Wagner@unimedizin-mainz.de>
Gesendet: Freitag, 10. September 2021 11:01
An: Schroeder, Thomas <Thomas.Schroeder@uk-essen.de>
Betreff: AW: CPX-351 Paper Bitte um kurzfristige Rückmeldung

Please request agreement from all authors including additions and deletions, these can be collected in the following way:

Email your co-authors with the change, and ask them to reply to your email confirming that they agree to these changes. Once you have collected these replies, please combine all of the co-authors’ email responses in one document and upload this file to your submission.

Dear Thomas,
I agree.

Kind regards

Eva

Dr. Eva Maria Wagner-Drouet
Leitung Stammzelltransplantation und zelluläre Imuntherapie
Hämatologie, internistische Onkologie und Pneumologie
3. Medizinische Klinik und Poliklinik
UCT der Universitätsmedizin Mainz
Tel: 06131-17-2712
Fax: 06131-17-472712

Sekretariat:
Frau I. Schmidt
Tel: 06131-17-5051
Mail: inge.schmidt@unimedizin-mainz.de

Von: Kobbe@med.uni-duesseldorf.de <Kobbe@med.uni-duesseldorf.de>
Gesendet: Freitag, 10. September 2021 10:41
An: Schroeder, Thomas <Thomas.Schroeder@uk-essen.de>; Hanoun, Maher <Maher.Hanoun@uk-essen.de>; Germing@med.uni-duesseldorf.de; Haas@med.uni-duesseldorf.de; Matthias.Stelljes@ukmuenster.de; lauseker@ibe.med.uni-muenchen.de; Johannes.Schetelig@uniklinikum-dresden.de; martin.bornhaeuser@uniklinikum-dresden.de; Christoph.Schliemann@ukmuenster.de; tim.sauer@med.uni-heidelberg.de; heuser.michael@mh-hannover.de; Hartmut.Doehner@uniklinik-ulm.de; Verena.Gaidzik@uniklinik-ulm.de; thol.felicitas@mh-hannover.de; tobias.holderried@ukbonn.de; eva.wagner@unimedizin-mainz.de; oliver.kriege@unimedizin-mainz.de; katharina.goetze@mri.tum.de; mareike.verbeek@mri.tum.de; a.morgner@skc.de; Konstanze.Doehner@uniklinik-ulm.de; uwe.platzbecker@medizin.uni-leipzig.de; Vucinic@medizin.uni-leipzig.de; nael.alakel@uniklinikum-dresden.de; katja.sockel@ukdd.de; william.krueger@uni-greifswald.de; stefan.krause@uk-erlangen.de; udo.holtick@uk-koeln.de; Kerstin.Schaefer-Eckart@klinikum-nuernberg.de; Josephine.Schroeder@helios-gesundheit.de; innere4@uk-halle.de; Lars.Fransecky@uksh.de; kmt-anfrage@ukw.de; med2@bernward-khs.de; charlotte.neuerburg@ukbonn.de; Sebastian.Scholl@med.uni-jena.de; c.jehn@asklepios.com; nadine.scheer-wiaczka@gk.de; jens.chemnitz@gk.de; m.haenel@skc.de; julia.unglaub@med.uni-heidelberg.de; Germing@med.uni-duesseldorf.de; Matthias.Stelljes@ukmuenster.de; Henning, Lea <Lea.Henning@uk-essen.de>
Cc: Rautenberg, Christina <Christina.Rautenberg@uk-essen.de>; Friedrich.Stoelzel@uniklinikum-dresden.de; JanMoritz.Middeke@uniklinikum-dresden.de
Betreff: AW: [Extern] - CPX-351 Paper Bitte um kurzfristige Rückmeldung

Please request agreement from all authors including additions and deletions, these can be collected in the following way:
Email your co-authors with the change, and ask them to reply to your email confirming that they agree to these changes. Once you have collected these replies, please combine all of the co-authors’ email responses in one document and upload this file to your submission.

Agreed, best GK

Professor Dr. med. Guido Kobbe
Oberarzt und Leiter der Stammzelltransplantation
Klinik für Hämatologie, Onkologie und Klinische Immunologie

Universitätsklinikum Düsseldorf
Gebäude 11.64
Moorenstr. 5
40225 Düsseldorf

Tel: *+49 211 81-16826*
Fax: *+49 211 81-17959*
e-Mail: kobbe@med.uni-duesseldorf.de
Internet: www.uniklinik-duesseldorf.de

Universitätsklinikum Düsseldorf (UKD) - Anstalt des öffentlichen Rechts
Moorenstraße 5 | 40225 Düsseldorf | Telefon: +49 211 81-00 | Telefax: +49 211 81-04855
Internet: www.uniklinik-duesseldorf.de | E-Mail: info@uniklinik-duesseldorf.de

Kommissarische Aufsichtsratsvorsitzende: Prof. Dr. Anja Steinbeck
Vorstand: Prof. Dr. Dr. Frank Schneider (Ärztlicher Direktor, Vorstandsvorsitzender) | Ekkehard Zimmer (Kaufmännischer Direktor, Stv. Vorstandsvorsitzender) | Prof. Dr. Nikolaj Klöcker (Dekan der Medizinischen Fakultät) | Torsten Rantzsch (Pflegedirektor) | Prof. Dr. Benedikt Pannen (Stv. Ärztlicher Direktor)

Steuernummer: 106-5773-0045 | IK: 26051 00 18
Bankverbindung: Stadtsparkasse Düsseldorf | BIC: DUSSDEDDXXX | IBAN: DE 94 3005 0110 0010 001550

Von: Konstanze.Doehner@uniklinik-ulm.de <Konstanze.Doehner@uniklinik-ulm.de>
Gesendet: Freitag, 10. September 2021 09:23
An: Schroeder, Thomas <Thomas.Schroeder@uk-essen.de>; Kobbe@med.uni-duesseldorf.de; Hanoun, Maher <Maher.Hanoun@uk-essen.de>; Germing@med.uni-duesseldorf.de; Haas@med.uni-duesseldorf.de; Matthias.Stelljes@ukmuenster.de; lauseker@ibe.med.uni-muenchen.de; Johannes.Schetelig@uniklinikum-dresden.de; martin.bornhaeuser@uniklinikum-dresden.de; Christoph.Schliemann@ukmuenster.de; tim.sauer@med.uni-heidelberg.de; heuser.michael@mh-hannover.de; Hartmut.Doehner@uniklinik-ulm.de; Verena.Gaidzik@uniklinik-ulm.de; thol.felicitas@mh-hannover.de; tobias.holderried@ukbonn.de; eva.wagner@unimedizin-mainz.de; oliver.kriege@unimedizin-mainz.de; katharina.goetze@mri.tum.de; mareike.verbeek@mri.tum.de; a.morgner@skc.de; uwe.platzbecker@medizin.uni-leipzig.de; Vucinic@medizin.uni-leipzig.de; nael.alakel@uniklinikum-dresden.de; katja.sockel@ukdd.de; william.krueger@uni-greifswald.de; stefan.krause@uk-erlangen.de; udo.holtick@uk-koeln.de; Kerstin.Schaefer-Eckart@klinikum-nuernberg.de; Josephine.Schroeder@helios-gesundheit.de; innere4@uk-halle.de; Lars.Fransecky@uksh.de; kmt-anfrage@ukw.de; med2@bernward-khs.de; charlotte.neuerburg@ukbonn.de; Sebastian.Scholl@med.uni-jena.de; c.jehn@asklepios.com; nadine.scheer-wiaczka@gk.de; jens.chemnitz@gk.de; m.haenel@skc.de; julia.unglaub@med.uni-heidelberg.de; Germing@med.uni-duesseldorf.de; Matthias.Stelljes@ukmuenster.de; Henning, Lea <Lea.Henning@uk-essen.de>
Cc: Rautenberg, Christina <Christina.Rautenberg@uk-essen.de>; Friedrich.Stoelzel@uniklinikum-dresden.de; JanMoritz.Middeke@uniklinikum-dresden.de
Betreff: AW: CPX-351 Paper Bitte um kurzfristige Rückmeldung

Please request agreement from all authors including additions and deletions, these can be collected in the following way:
Email your co-authors with the change, and ask them to reply to your email confirming that

they agree to these changes. Once you have collected these replies, please combine all of the co-authors' email responses in one document and upload this file to your submission.

Agreed!
Konstanze Döhner

Prof. Dr. Konstanze Döhner
University Hospital of Ulm
Department of Internal Medicine III
Albert-Einstein-Allee 23
89081 Ulm
Germany
Phone: +49-731-50045543
FAX: +49-731-50045505
email: konstanze.doehner@uniklinik-ulm.de

Von: Thol.Felicitas@mh-hannover.de <Thol.Felicitas@mh-hannover.de>
Gesendet: Freitag, 10. September 2021 09:17
An: Schroeder, Thomas <Thomas.Schroeder@uk-essen.de>
Betreff: AW: CPX-351 Paper Bitte um kurzfristige Rückmeldung

Please request agreement from all authors including additions and deletions, these can be collected in the following way:
Email your co-authors with the change, and ask them to reply to your email confirming that they agree to these changes. Once you have collected these replies, please combine all of the co-authors' email responses in one document and upload this file to your submission.

Lieber Thomas,
Agreed.
Viele Grüße
Felicitas

Von: Sauer, Tim <Tim.Sauer@med.uni-heidelberg.de>
Gesendet: Freitag, 10. September 2021 09:15
An: Schroeder, Thomas <Thomas.Schroeder@uk-essen.de>
Betreff: AW: CPX-351 Paper Bitte um kurzfristige Rückmeldung
Please request agreement from all authors including additions and deletions, these can be collected in the following way:
Email your co-authors with the change, and ask them to reply to your email confirming that they agree to these changes. Once you have collected these replies, please combine all of the co-authors' email responses in one document and upload this file to your submission.

Agreed.

Best regards,

Tim Sauer

Dr. med. Tim Sauer
Oberarzt | Taskforce-Leiter Akute myeloische Leukämie (AML)
und myeloproliferative Neoplasien (MPN)
Medizinische Klinik (Krehl-Klinik) | Zentrum für Innere Medizin
Klinik für Hämatologie, Onkologie und Rheumatologie | Innere Medizin V

Universitätsklinikum Heidelberg | Im Neuenheimer Feld 410 | 69120 Heidelberg
Tel.: +49 6221 56-38010 | Fax: +49 6221 56-5435 | E-Mail: tim.sauer@med.uni-heidelberg.de http://www.klinikum.uni-heidelberg.de

Von: Hartmut.Doehner@uniklinik-ulm.de <Hartmut.Doehner@uniklinik-ulm.de>
Gesendet: Freitag, 10. September 2021 09:04
An: Schroeder, Thomas <Thomas.Schroeder@uk-essen.de>
Betreff: AW: CPX-351 Paper Bitte um kurzfristige Rückmeldung
Please request agreement from all authors including additions and deletions, these can be collected in the following way:
Email your co-authors with the change, and ask them to reply to your email confirming that they agree to these changes. Once you have collected these replies, please combine all of the co-authors' email responses in one document and upload this file to your submission.

Agreed.

Viele Grüße, Hartmut Döhner

Von: Morgner, Anke OÄ Dr. med. <a.morgner@skc.de>
Gesendet: Freitag, 10. September 2021 08:39
An: Schroeder, Thomas <Thomas.Schroeder@uk-essen.de>
Betreff: AW: CPX-351 Paper Bitte um kurzfristige Rückmeldung

Please request agreement from all authors including additions and deletions, these can be collected in the following way:
Email your co-authors with the change, and ask them to reply to your email confirming that they agree to these changes. Once you have collected these replies, please combine all of the co-authors' email responses in one document and upload this file to your submission.

Agreed

Best regards.

A.morgner

Von: Scholl, Sebastian <Sebastian.Scholl@med.uni-jena.de>
Gesendet: Freitag, 10. September 2021 08:30
An: Schroeder, Thomas <Thomas.Schroeder@uk-essen.de>
Betreff: AW: CPX-351 Paper Bitte um kurzfristige Rückmeldung

Please request agreement from all authors including additions and deletions, these can be collected in the following way:
Email your co-authors with the change, and ask them to reply to your email confirming that they agree to these changes. Once you have collected these replies, please combine all of the co-authors' email responses in one document and upload this file to your submission.

„Agreed"

Herzlichen Dank und viele Grüße
Sebastian Scholl

Von: Haas@med.uni-duesseldorf.de <Haas@med.uni-duesseldorf.de>
Gesendet: Freitag, 10. September 2021 08:29
An: Mareike.Verbeek@mri.tum.de; Schroeder, Thomas <Thomas.Schroeder@uk-essen.de>; Kobbe@med.uni-duesseldorf.de; Hanoun, Maher <Maher.Hanoun@uk-essen.de>; Germing@med.uni-duesseldorf.de; Matthias.Stelljes@ukmuenster.de; lauseker@ibe.med.uni-muenchen.de; Johannes.Schetelig@uniklinikum-dresden.de; martin.bornhaeuser@uniklinikum-dresden.de; Christoph.Schliemann@ukmuenster.de; tim.sauer@med.uni-heidelberg.de; heuser.michael@mh-hannover.de; Hartmut.Doehner@uniklinik-ulm.de; Verena.Gaidzik@uniklinik-ulm.de; thol.felicitas@mh-hannover.de; tobias.holderried@ukbonn.de; eva.wagner@unimedizin-mainz.de; oliver.kriege@unimedizin-mainz.de; Katharina.Goetze@mri.tum.de; a.morgner@skc.de; Konstanze.Doehner@uniklinik-ulm.de; uwe.platzbecker@medizin.uni-leipzig.de; Vucinic@medizin.uni-leipzig.de; nael.alakel@uniklinikum-dresden.de; katja.sockel@ukdd.de; william.krueger@uni-greifswald.de; stefan.krause@uk-erlangen.de; udo.holtick@uk-koeln.de; Kerstin.Schaefer-Eckart@klinikum-nuernberg.de; Josephine.Schroeder@helios-gesundheit.de; innere4@uk-halle.de; Lars.Fransecky@uksh.de; kmt-anfrage@ukw.de; med2@bernward-khs.de; charlotte.neuerburg@ukbonn.de; Sebastian.Scholl@med.uni-jena.de; c.jehn@asklepios.com; nadine.scheer-wiaczka@gk.de; jens.chemnitz@gk.de; m.haenel@skc.de; julia.unglaub@med.uni-heidelberg.de; Germing@med.uni-duesseldorf.de; Matthias.Stelljes@ukmuenster.de; Henning, Lea <Lea.Henning@uk-essen.de>
Cc: Rautenberg, Christina <Christina.Rautenberg@uk-essen.de>; Friedrich.Stoelzel@uniklinikum-dresden.de; JanMoritz.Middeke@uniklinikum-dresden.de
Betreff: AW: [Extern] - AW: CPX-351 Paper Bitte um kurzfristige Rückmeldung

Please request agreement from all authors including additions and deletions, these can be collected in the following way:
Email your co-authors with the change, and ask them to reply to your email confirming that they agree to these changes. Once you have collected these replies, please combine all of the co-authors' email responses in one document and upload this file to your submission.

Agreed!
With kind regards
Rainer Haas

Von: Verbeek, Mareike <Mareike.Verbeek@mri.tum.de>
Gesendet: Freitag, 10. September 2021 08:23
An: Schroeder, Thomas <Thomas.Schroeder@uk-essen.de>; 'Kobbe@med.uni-duesseldorf.de' <Kobbe@med.uni-duesseldorf.de>; Hanoun, Maher <Maher.Hanoun@uk-essen.de>; 'Germing@med.uni-duesseldorf.de' <Germing@med.uni-duesseldorf.de>; 'Haas@med.uni-duesseldorf.de' <Haas@med.uni-duesseldorf.de>; 'Matthias.Stelljes@ukmuenster.de' <Matthias.Stelljes@ukmuenster.de>; 'lauseker@ibe.med.uni-muenchen.de' <lauseker@ibe.med.uni-muenchen.de>; 'Schetelig, Johannes' <Johannes.Schetelig@uniklinikum-dresden.de>; 'martin.bornhaeuser@uniklinikum-dresden.de' <martin.bornhaeuser@uniklinikum-dresden.de>; 'Christoph.Schliemann@ukmuenster.de' <Christoph.Schliemann@ukmuenster.de>; 'tim.sauer@med.uni-heidelberg.de'

<tim.sauer@med.uni-heidelberg.de>; 'heuser.michael@mh-hannover.de' <heuser.michael@mh-hannover.de>; 'Döhner Hartmut' <Hartmut.Doehner@uniklinik-ulm.de>; 'Gaidzik Verena' <Verena.Gaidzik@uniklinik-ulm.de>; 'thol.felicitas@mh-hannover.de' <thol.felicitas@mh-hannover.de>; 'tobias.holderried@ukbonn.de' <tobias.holderried@ukbonn.de>; 'eva.wagner@unimedizin-mainz.de' <eva.wagner@unimedizin-mainz.de>; 'oliver.kriege@unimedizin-mainz.de' <oliver.kriege@unimedizin-mainz.de>; Götze, Katharina <Katharina.Goetze@mri.tum.de>; 'a.morgner@skc.de' <a.morgner@skc.de>; 'Döhner Konstanze' <Konstanze.Doehner@uniklinik-ulm.de>; 'uwe.platzbecker@medizin.uni-leipzig.de' <uwe.platzbecker@medizin.uni-leipzig.de>; 'Vladan' <Vucinic@medizin.uni-leipzig.de>; 'nael.alakel@uniklinikum-dresden.de' <nael.alakel@uniklinikum-dresden.de>; 'katja.sockel@ukdd.de' <katja.sockel@ukdd.de>; 'william.krueger@uni-greifswald.de' <william.krueger@uni-greifswald.de>; 'stefan.krause@uk-erlangen.de' <stefan.krause@uk-erlangen.de>; 'udo.holtick@uk-koeln.de' <udo.holtick@uk-koeln.de>; 'Kerstin.Schaefer-Eckart@klinikum-nuernberg.de' <Kerstin.Schaefer-Eckart@klinikum-nuernberg.de>; 'Josephine.Schroeder@helios-gesundheit.de' <Josephine.Schroeder@helios-gesundheit.de>; 'innere4@uk-halle.de' <innere4@uk-halle.de>; 'Lars.Fransecky@uksh.de' <Lars.Fransecky@uksh.de>; 'kmt-anfrage@ukw.de' <kmt-anfrage@ukw.de>; 'med2@bernward-khs.de' <med2@bernward-khs.de>; 'charlotte.neuerburg@ukbonn.de' <charlotte.neuerburg@ukbonn.de>; 'Sebastian.Scholl@med.uni-jena.de' <Sebastian.Scholl@med.uni-jena.de>; 'c.jehn@asklepios.com' <c.jehn@asklepios.com>; 'nadine.scheer-wiaczka@gk.de' <nadine.scheer-wiaczka@gk.de>; 'jens.chemnitz@gk.de' <jens.chemnitz@gk.de>; 'm.haenel@skc.de' <m.haenel@skc.de>; 'julia.unglaub@med.uni-heidelberg.de' <julia.unglaub@med.uni-heidelberg.de>; 'Germing@med.uni-duesseldorf.de' <Germing@med.uni-duesseldorf.de>; 'Matthias.Stelljes@ukmuenster.de' <Matthias.Stelljes@ukmuenster.de>; Henning, Lea <Lea.Henning@uk-essen.de>
Cc: Rautenberg, Christina <Christina.Rautenberg@uk-essen.de>; 'Stölzel, Friedrich' <Friedrich.Stoelzel@uniklinikum-dresden.de>; 'Middeke, Jan Moritz' <JanMoritz.Middeke@uniklinikum-dresden.de>
Betreff: AW: CPX-351 Paper Bitte um kurzfristige Rückmeldung

Please request agreement from all authors including additions and deletions, these can be collected in the following way:
Email your co-authors with the change, and ask them to reply to your email confirming that they agree to these changes. Once you have collected these replies, please combine all of the co-authors’ email responses in one document and upload this file to your submission.

Agreed!

Best
Mareike

Von: Unglaub, Julia <Julia.Unglaub@med.uni-heidelberg.de>
Gesendet: Freitag, 10. September 2021 08:21
An: Schroeder, Thomas <Thomas.Schroeder@uk-essen.de>; 'Kobbe@med.uni-duesseldorf.de' <Kobbe@med.uni-duesseldorf.de>; Hanoun, Maher <Maher.Hanoun@uk-essen.de>; 'Germing@med.uni-duesseldorf.de' <Germing@med.uni-duesseldorf.de>; 'Haas@med.uni-duesseldorf.de' <Haas@med.uni-duesseldorf.de>; 'Matthias.Stelljes@ukmuenster.de' <Matthias.Stelljes@ukmuenster.de>; 'lauseker@ibe.med.uni-muenchen.de' <lauseker@ibe.med.uni-muenchen.de>; 'Schetelig,

Johannes' <Johannes.Schetelig@uniklinikum-dresden.de>; 'martin.bornhaeuser@uniklinikum-dresden.de' <martin.bornhaeuser@uniklinikum-dresden.de>; 'Christoph.Schliemann@ukmuenster.de' <Christoph.Schliemann@ukmuenster.de>; Sauer, Tim <Tim.Sauer@med.uni-heidelberg.de>; 'heuser.michael@mh-hannover.de' <heuser.michael@mh-hannover.de>; 'Döhner Hartmut' <Hartmut.Doehner@uniklinik-ulm.de>; 'Gaidzik Verena' <Verena.Gaidzik@uniklinik-ulm.de>; 'thol.felicitas@mh-hannover.de' <thol.felicitas@mh-hannover.de>; 'tobias.holderried@ukbonn.de' <tobias.holderried@ukbonn.de>; 'eva.wagner@unimedizin-mainz.de' <eva.wagner@unimedizin-mainz.de>; 'oliver.kriege@unimedizin-mainz.de' <oliver.kriege@unimedizin-mainz.de>; 'katharina.goetze@mri.tum.de' <katharina.goetze@mri.tum.de>; 'mareike.verbeek@mri.tum.de' <mareike.verbeek@mri.tum.de>; 'a.morgner@skc.de' <a.morgner@skc.de>; 'Döhner Konstanze' <Konstanze.Doehner@uniklinik-ulm.de>; 'uwe.platzbecker@medizin.uni-leipzig.de' <uwe.platzbecker@medizin.uni-leipzig.de>; 'Vladan' <Vucinic@medizin.uni-leipzig.de>; 'nael.alakel@uniklinikum-dresden.de' <nael.alakel@uniklinikum-dresden.de>; 'katja.sockel@ukdd.de' <katja.sockel@ukdd.de>; 'william.krueger@uni-greifswald.de' <william.krueger@uni-greifswald.de>; 'stefan.krause@uk-erlangen.de' <stefan.krause@uk-erlangen.de>; 'udo.holtick@uk-koeln.de' <udo.holtick@uk-koeln.de>; 'Kerstin.Schaefer-Eckart@klinikum-nuernberg.de' <Kerstin.Schaefer-Eckart@klinikum-nuernberg.de>; 'Josephine.Schroeder@helios-gesundheit.de' <Josephine.Schroeder@helios-gesundheit.de>; 'innere4@uk-halle.de' <innere4@uk-halle.de>; 'Lars.Fransecky@uksh.de' <Lars.Fransecky@uksh.de>; 'kmt-anfrage@ukw.de' <kmt-anfrage@ukw.de>; 'med2@bernward-khs.de' <med2@bernward-khs.de>; 'charlotte.neuerburg@ukbonn.de' <charlotte.neuerburg@ukbonn.de>; 'Sebastian.Scholl@med.uni-jena.de' <Sebastian.Scholl@med.uni-jena.de>; 'c.jehn@asklepios.com' <c.jehn@asklepios.com>; 'nadine.scheer-wiaczka@gk.de' <nadine.scheer-wiaczka@gk.de>; 'jens.chemnitz@gk.de' <jens.chemnitz@gk.de>; 'm.haenel@skc.de' <m.haenel@skc.de>; 'Germing@med.uni-duesseldorf.de' <Germing@med.uni-duesseldorf.de>; 'Matthias.Stelljes@ukmuenster.de' <Matthias.Stelljes@ukmuenster.de>; Henning, Lea <Lea.Henning@uk-essen.de>
Cc: Rautenberg, Christina <Christina.Rautenberg@uk-essen.de>; 'Stölzel, Friedrich' <Friedrich.Stoelzel@uniklinikum-dresden.de>; 'Middeke, Jan Moritz' <JanMoritz.Middeke@uniklinikum-dresden.de>
Betreff: AW: CPX-351 Paper Bitte um kurzfristige Rückmeldung

Please request agreement from all authors including additions and deletions, these can be collected in the following way:
Email your co-authors with the change, and ask them to reply to your email confirming that they agree to these changes. Once you have collected these replies, please combine all of the co-authors’ email responses in one document and upload this file to your submission.

Agreed!

Kind regards
Julia Unglaub

Von: Kriege, Oliver <Oliver.Kriege@unimedizin-mainz.de>
Gesendet: Freitag, 10. September 2021 08:05
An: Schroeder, Thomas <Thomas.Schroeder@uk-essen.de>
Betreff: AW: CPX-351 Paper Bitte um kurzfristige Rückmeldung

Please request agreement from all authors including additions and deletions, these can be collected in the following way:
Email your co-authors with the change, and ask them to reply to your email confirming that they agree to these changes. Once you have collected these replies, please combine all of the co-authors' email responses in one document and upload this file to your submission.

Agreed

Lieben Gruß
Oliver Kriege

Dr. med. O. Kriege
Facharzt Innere Medizin, Hämatologie und Onkologie
III. Medizinische Klinik und Poliklinik
Hämatologie, internistische Onkologie, Pneumologie
UCT der Universitätsmedizin Mainz

Tel: 06131-17-6322
Fax: 06131-17-3449
Funk: 194-6790
Mail: oliver.kriege@unimedizin-mainz.de

JG|U UNIVERSITÄTSmedizin. MAINZ

Von: Krause, Stefan <Stefan.Krause@uk-erlangen.de>
Gesendet: Freitag, 10. September 2021 07:53
An: Schroeder, Thomas <Thomas.Schroeder@uk-essen.de>
Betreff: AW: CPX-351 Paper Bitte um kurzfristige Rückmeldung

Please request agreement from all authors including additions and deletions, these can be collected in the following way:
Email your co-authors with the change, and ask them to reply to your email confirming that they agree to these changes. Once you have collected these replies, please combine all of the co-authors' email responses in one document and upload this file to your submission.

agreed

Mit vielen Grüßen
Stefan Krause
--
Prof. Dr. Stefan W. Krause, leitender Oberarzt
Medizinische Klinik 5 - Hämatologie/Onkologie
Universitätsklinik, Ulmenweg 18, 91054 Erlangen
ph: 09131 85-35957 fax: 09131 85-35958

Von: Germing@med.uni-duesseldorf.de <Germing@med.uni-duesseldorf.de>
Gesendet: Freitag, 10. September 2021 07:49
An: Schroeder, Thomas <Thomas.Schroeder@uk-essen.de>; Kobbe@med.uni-duesseldorf.de; Hanoun, Maher <Maher.Hanoun@uk-essen.de>; Haas@med.uni-duesseldorf.de; Matthias.Stelljes@ukmuenster.de; lauseker@ibe.med.uni-muenchen.de; Johannes.Schetelig@uniklinikum-dresden.de; martin.bornhaeuser@uniklinikum-dresden.de; Christoph.Schliemann@ukmuenster.de; tim.sauer@med.uni-heidelberg.de;

heuser.michael@mh-hannover.de; Hartmut.Doehner@uniklinik-ulm.de; Verena.Gaidzik@uniklinik-ulm.de; thol.felicitas@mh-hannover.de; tobias.holderried@ukbonn.de; eva.wagner@unimedizin-mainz.de; oliver.kriege@unimedizin-mainz.de; katharina.goetze@mri.tum.de; mareike.verbeek@mri.tum.de; a.morgner@skc.de; Konstanze.Doehner@uniklinik-ulm.de; uwe.platzbecker@medizin.uni-leipzig.de; Vucinic@medizin.uni-leipzig.de; nael.alakel@uniklinikum-dresden.de; katja.sockel@ukdd.de; william.krueger@uni-greifswald.de; stefan.krause@uk-erlangen.de; udo.holtick@uk-koeln.de; Kerstin.Schaefer-Eckart@klinikum-nuernberg.de; Josephine.Schroeder@helios-gesundheit.de; innere4@uk-halle.de; Lars.Fransecky@uksh.de; kmt-anfrage@ukw.de; med2@bernward-khs.de; charlotte.neuerburg@ukbonn.de; Sebastian.Scholl@med.uni-jena.de; c.jehn@asklepios.com; nadine.scheer-wiaczka@gk.de; jens.chemnitz@gk.de; m.haenel@skc.de; julia.unglaub@med.uni-heidelberg.de; Matthias.Stelljes@ukmuenster.de; Henning, Lea <Lea.Henning@uk-essen.de>
Cc: Rautenberg, Christina <Christina.Rautenberg@uk-essen.de>; Friedrich.Stoelzel@uniklinikum-dresden.de; JanMoritz.Middeke@uniklinikum-dresden.de
Betreff: AW: [Extern] - CPX-351 Paper Bitte um kurzfristige Rückmeldung

Please request agreement from all authors including additions and deletions, these can be collected in the following way:
Email your co-authors with the change, and ask them to reply to your email confirming that they agree to these changes. Once you have collected these replies, please combine all of the co-authors’ email responses in one document and upload this file to your submission.

Agreed!
Best
Ulrich

Von: Hanoun, Maher <Maher.Hanoun@uk-essen.de>
Gesendet: Freitag, 10. September 2021 07:06
An: Schroeder, Thomas <Thomas.Schroeder@uk-essen.de>
Betreff: AW: CPX-351 Paper Bitte um kurzfristige Rückmeldung

Please request agreement from all authors including additions and deletions, these can be collected in the following way:
Email your co-authors with the change, and ask them to reply to your email confirming that they agree to these changes. Once you have collected these replies, please combine all of the co-authors’ email responses in one document and upload this file to your submission.

Agreed!

Von: Matthias.Stelljes@ukmuenster.de <Matthias.Stelljes@ukmuenster.de>
Gesendet: Donnerstag, 9. September 2021 20:30
An: Schroeder, Thomas <Thomas.Schroeder@uk-essen.de>
Betreff: Re: CPX-351 Paper Bitte um kurzfristige Rückmeldung

Please request agreement from all authors including additions and deletions, these can be collected in the following way:
Email your co-authors with the change, and ask them to reply to your email confirming that they agree to these changes. Once you have collected these replies, please combine all of the co-authors’ email responses in one document and upload this file to your submission.

Agreed

Mit freundlichen Grüßen

M. Stelljes

Von meinem Mobilgerät gesendet

Von: Vucinic, Vladan <Vladan.Vucinic@medizin.uni-leipzig.de>
Gesendet: Donnerstag, 9. September 2021 20:34
An: Schroeder, Thomas <Thomas.Schroeder@uk-essen.de>
Cc: Rautenberg, Christina <Christina.Rautenberg@uk-essen.de>
Betreff: AW: CPX-351 Paper Bitte um kurzfristige Rückmeldung

Lieber Herr Schroeder,

in der Sammel E-mail gab es tatsächlich ein Fehler bezogen auf meine E-mail Adresse. Selbstverständlich bin ich einverstanden.

Dear all,

of course I agree.

Please request agreement from all authors including additions and deletions, these can be collected in the following way:
Email your co-authors with the change, and ask them to reply to your email confirming that they agree to these changes. Once you have collected these replies, please combine all of the co-authors' email responses in one document and upload this file to your submission.

Best,
Vladan Vucinic

Von: Christoph.Schliemann@ukmuenster.de <Christoph.Schliemann@ukmuenster.de>
Gesendet: Donnerstag, 9. September 2021 20:35
An: Schroeder, Thomas <Thomas.Schroeder@uk-essen.de>
Betreff: Re: CPX-351 Paper Bitte um kurzfristige Rückmeldung

Please request agreement from all authors including additions and deletions, these can be collected in the following way:
Email your co-authors with the change, and ask them to reply to your email confirming that they agree to these changes. Once you have collected these replies, please combine all of the co-authors' email responses in one document and upload this file to your submission.

I agree

Best,

Christoph Schliemann

Von: Tobias.Holderried@ukbonn.de <Tobias.Holderried@ukbonn.de>
Gesendet: Donnerstag, 9. September 2021 20:50
An: Schroeder, Thomas <Thomas.Schroeder@uk-essen.de>
Betreff: Re: CPX-351 Paper Bitte um kurzfristige Rückmeldung

Agreed.

Please request agreement from all authors including additions and deletions, these can be collected in the following way:
Email your co-authors with the change, and ask them to reply to your email confirming that they agree to these changes. Once you have collected these replies, please combine all of the co-authors' email responses in one document and upload this file to your submission.

Von: Stölzel, Friedrich <Friedrich.Stoelzel@uniklinikum-dresden.de>
Gesendet: Donnerstag, 9. September 2021 21:01
An: Schroeder, Thomas <Thomas.Schroeder@uk-essen.de>
Betreff: AW: CPX-351 Paper Bitte um kurzfristige Rückmeldung
Please request agreement from all authors including additions and deletions, these can be collected in the following way:
Email your co-authors with the change, and ask them to reply to your email confirming that they agree to these changes. Once you have collected these replies, please combine all of the co-authors' email responses in one document and upload this file to your submission.

Agreed,
Friedrich Stölzel

Von: Verena.Gaidzik@uniklinik-ulm.de <Verena.Gaidzik@uniklinik-ulm.de>
Gesendet: Donnerstag, 9. September 2021 21:04
An: Schroeder, Thomas <Thomas.Schroeder@uk-essen.de>
Betreff: AW: CPX-351 Paper Bitte um kurzfristige Rückmeldung

Please request agreement from all authors including additions and deletions, these can be collected in the following way:
Email your co-authors with the change, and ask them to reply to your email confirming that they agree to these changes. Once you have collected these replies, please combine all of the co-authors' email responses in one document and upload this file to your submission.

Lieber Herr Schroeder,

agreed!

Herzlichen Glückwunsch und vielen Dank für die Berücksichtigung als Koautorin.
Viele Grüße,
Verena Gaidzik

Von: Chemnitz, Jens < jens.chemnitz@gk.de>
Gesendet: Donnerstag, 9. September 2021 22:02
An: Schroeder, Thomas <Thomas.Schroeder@uk-essen.de>
Betreff: AW: CPX-351 Paper Bitte um kurzfristige Rückmeldung

Agreed

Please request agreement from all authors including additions and deletions, these can be collected in the following way:
Email your co-authors with the change, and ask them to reply to your email confirming that they agree to these changes. Once you have collected these replies, please combine all of the co-authors’ email responses in one document and upload this file to your submission.

Jens Chemnitz

Von: Udo Holtick <udo.holtick@uk-koeln.de>
Gesendet: Donnerstag, 9. September 2021 21:06
An: Schroeder, Thomas <Thomas.Schroeder@uk-essen.de>
Betreff: RE: CPX-351 Paper Bitte um kurzfristige Rückmeldung
Priorität: Hoch

Agreed.
Kind regards,
Udo

Please request agreement from all authors including additions and deletions, these can be collected in the following way:
Email your co-authors with the change, and ask them to reply to your email confirming that they agree to these changes. Once you have collected these replies, please combine all of the co-authors’ email responses in one document and upload this file to your submission.

Von: Götze, Katharina <katharina.goetze@tum.de>
Gesendet: Donnerstag, 9. September 2021 21:33
An: Schroeder, Thomas <Thomas.Schroeder@uk-essen.de>
Betreff: Re: CPX-351 Paper Bitte um kurzfristige Rückmeldung

Please request agreement from all authors including additions and deletions, these can be collected in the following way:
Email your co-authors with the change, and ask them to reply to your email confirming that they agree to these changes. Once you have collected these replies, please combine all of the co-authors’ email responses in one document and upload this file to your submission.

agreed!

Sent from my iPhone

Von: Wass, Maxi < maxi.wass@uk-halle.de>
Gesendet: Donnerstag, 9. September 2021 23:02
An: Schroeder, Thomas <Thomas.Schroeder@uk-essen.de>
Betreff: AW: CPX-351 Paper Bitte um kurzfristige Rückmeldung

Agreed

Please request agreement from all authors including additions and deletions, these can be collected in the following way:
Email your co-authors with the change, and ask them to reply to your email confirming that they agree to these changes. Once you have collected these replies, please combine all of the co-authors' email responses in one document and upload this file to your submission.

Maxi Wass

Von: Heuser.Michael@mh-hannover.de <Heuser.Michael@mh-hannover.de>
Gesendet: Donnerstag, 9. September 2021 21:39
An: Schroeder, Thomas <Thomas.Schroeder@uk-essen.de>
Betreff: AW: CPX-351 Paper Bitte um kurzfristige Rückmeldung

Please request agreement from all authors including additions and deletions, these can be collected in the following way:
Email your co-authors with the change, and ask them to reply to your email confirming that they agree to these changes. Once you have collected these replies, please combine all of the co-authors' email responses in one document and upload this file to your submission.

Dear Thomas,

I agree.

Kind regards,

Michael

Von: Koch, Katrin <Katrin.Koch@mri.tum.de>
Gesendet: Donnerstag, 9. September 2021 21:47
An: Schroeder, Thomas <Thomas.Schroeder@uk-essen.de>
Cc: Götze, Katharina TUM <katharina.goetze@tum.de>
Betreff: AW: CPX-351 Paper Bitte um kurzfristige Rückmeldung

Agreed
Please request agreement from all authors including additions and deletions, these can be collected in the following way:
Email your co-authors with the change, and ask them to reply to your email confirming that they agree to these changes. Once you have collected these replies, please combine all of the co-authors' email responses in one document and upload this file to your submission.

Lieber Herr Schroeder,

Vielen herzlichen Dank für die Ergänzung!

Viele Grüße
Katrin Koch

Klinikum rechts der Isar

Technische Universität München
III. Medizinische Klinik und Poliklinik
Ismaninger Str. 22
81675 München

Email: katrin.koch@mri.tum.de

Von: Sockel, Katja <Katja.Sockel@uniklinikum-dresden.de>
Gesendet: Donnerstag, 9. September 2021 22:07
An: Schroeder, Thomas <Thomas.Schroeder@uk-essen.de>
Betreff: AW: CPX-351 Paper Bitte um kurzfristige Rückmeldung

agreed, of course.

Please request agreement from all authors including additions and deletions, these can be collected in the following way:
Email your co-authors with the change, and ask them to reply to your email confirming that they agree to these changes. Once you have collected these replies, please combine all of the co-authors' email responses in one document and upload this file to your submission.

Dr.med.Katja Sockel

Funktionsoberärztin

Fachärztin für Innere Medizin/Hämatologie/Onkologie

Medizinische Klinik und Poliklinik I
Tel. +49 (0)351 458-15627

Fax. +49 (0)351 458-4373

Universitätsklinikum Carl Gustav Carus
an der Technischen Universität Dresden
Anstalt des öffentlichen Rechts des Freistaates Sachsen
Fetscherstraße 74, 01307 Dresden
http://www.uniklinikum-dresden.de<http://www.uniklinikum-dresden.de/

Von: Middeke, Jan Moritz <JanMoritz.Middeke@uniklinikum-dresden.de>
Gesendet: Donnerstag, 9. September 2021 22:24
An: Schroeder, Thomas <Thomas.Schroeder@uk-essen.de>
Betreff: AW: CPX-351 Paper Bitte um kurzfristige Rückmeldung

Please request agreement from all authors including additions and deletions, these can be collected in the following way:
Email your co-authors with the change, and ask them to reply to your email confirming that they agree to these changes. Once you have collected these replies, please combine all of the co-authors' email responses in one document and upload this file to your submission.

Agreed

Von: Hänel, Mathias ChA Priv.-Doz. Dr. med. <m.haenel@skc.de>
Gesendet: Freitag, 10. September 2021 06:12

An: Schroeder, Thomas <Thomas.Schroeder@uk-essen.de>
Betreff: AW: CPX-351 Paper Bitte um kurzfristige Rückmeldung
Please request agreement from all authors including additions and deletions, these can be collected in the following way:
Email your co-authors with the change, and ask them to reply to your email confirming that they agree to these changes. Once you have collected these replies, please combine all of the co-authors' email responses in one document and upload this file to your submission.

Agreed.

Von: Bornhäuser, Martin <Martin.Bornhaeuser@uniklinikum-dresden.de>
Gesendet: Freitag, 10. September 2021 00:44
An: Schroeder, Thomas <Thomas.Schroeder@uk-essen.de>
Betreff: Re: CPX-351 Paper Bitte um kurzfristige Rückmeldung
Please request agreement from all authors including additions and deletions, these can be collected in the following way:
Email your co-authors with the change, and ask them to reply to your email confirming that they agree to these changes. Once you have collected these replies, please combine all of the co-authors' email responses in one document and upload this file to your submission.

Agreed

Von: Röllig, Christoph <Christoph.Roellig@uniklinikum-dresden.de>
Gesendet: Donnerstag, 16. September 2021 07:34
An: Schroeder, Thomas <Thomas.Schroeder@uk-essen.de>
Betreff: AW: CPX-351 Paper Bitte um kurzfristige Rückmeldung

Please request agreement from all authors including additions and deletions, these can be collected in the following way:
Email your co-authors with the change, and ask them to reply to your email confirming that they agree to these changes. Once you have collected these replies, please combine all of the co-authors' email responses in one document and upload this file to your submission.

Agreed
Christoph Röllig

Von: Rautenberg, Christina <Christina.Rautenberg@uk-essen.de>
Gesendet: Donnerstag, 16. September 2021 07:38
An: Schroeder, Thomas <Thomas.Schroeder@uk-essen.de>
Betreff: AW: CPX-351 Paper Bitte um kurzfristige Rückmeldung

Agreed.

Christina Rautenberg

Please request agreement from all authors including additions and deletions, these can be collected in the following way:
Email your co-authors with the change, and ask them to reply to your email confirming that they agree to these changes. Once you have collected these replies, please combine all of the co-authors' email responses in one document and upload this file to your submission.

Von: Röhnert, Maximilian-Alexander <Maximilian-Alexander.Roehnert@uniklinikum-dresden.de>
Gesendet: Donnerstag, 16. September 2021 08:41
An: Schroeder, Thomas <Thomas.Schroeder@uk-essen.de>
Betreff: AW: CPX-351 Paper Bitte um kurzfristige Rückmeldung

Sehr geehrter Herr PD Dr. med. Schröder,

leider war meine E-Mail-Adresse nicht ganz korrekt, so dass ich erst nach Weiterleitung der E-Mail durch Friedrich Stölzle antworten kann:

Agreed - Maximilian Alexander Röhnert

Please request agreement from all authors including additions and deletions, these can be collected in the following way:
Email your co-authors with the change, and ask them to reply to your email confirming that they agree to these changes. Once you have collected these replies, please combine all of the co-authors' email responses in one document and upload this file to your submission.

Vielen Dank und viele Grüße

Max Röhnert

Von: Faltin, Josephine <Josephine.Faltin@helios-gesundheit.de>
Gesendet: Donnerstag, 16. September 2021 08:14
An: Schroeder, Thomas <Thomas.Schroeder@uk-essen.de>
Betreff: AW: CPX-351 Paper Bitte um kurzfristige Rückmeldung

Agreed

-----Ursprüngliche Nachricht-----
Von: Kraus, Sabrina <Kraus_S3@ukw.de>
Gesendet: Donnerstag, 16. September 2021 07:56
An: Schroeder, Thomas <Thomas.Schroeder@uk-essen.de>
Betreff: AW: [Ext] AW: CPX-351 Paper Bitte um kurzfristige Rückmeldung

Lieber Herr Schroeder,

herby I agree to these changes.
Please request agreement from all authors including additions and deletions, these can be collected in the following way:
Email your co-authors with the change, and ask them to reply to your email confirming that they agree to these changes. Once you have collected these replies, please combine all of the co-authors' email responses in one document and upload this file to your submission.

Best,

Sabrina Kraus

Von: Schaefer-Eckart, Kerstin Dr. <Kerstin.Schaefer-Eckart@klinikum-nuernberg.de>
Gesendet: Donnerstag, 16. September 2021 07:44
An: Schroeder, Thomas <Thomas.Schroeder@uk-essen.de>
Betreff: AW: CPX-351 Paper Bitte um kurzfristige Rückmeldung

Please request agreement from all authors including additions and deletions, these can be collected in the following way:
Email your co-authors with the change, and ask them to reply to your email confirming that they agree to these changes. Once you have collected these replies, please combine all of the co-authors' email responses in one document and upload this file to your submission.

Habe ich zwar schon ,aber ich mache es sicherheitshalber nochmal: agreed

Dr. K. Schäfer-Eckart
Leitende Oberärztin
Einheit für Knochenmarktransplantation
Klinik für Innere Medizin 5
Schwerpunkt Onkologie/Hämatologie
Universitätsklinik der Paracelsus Medizinischen Privatuniversität
Prof.-Ernst-Nathan-Str. 1, 90419 Nürnberg
T: +49 911 398-3650
F: +49 911 398-3657

GO GREEN
GO PAPERLESS
